# Supplementary material for: Astrocytic-OTUD7B ameliorates murine experimental autoimmune encephalomyelitis by stabilizing glial fibrillary acidic protein and preventing inflammation
Source: Nat Commun. 2025 Oct 20;16:9279. doi: 10.1038/s41467-025-65093-4 (PMC12537900; doi:10.1038/s41467-025-65093-4)

Supplementary figure 1

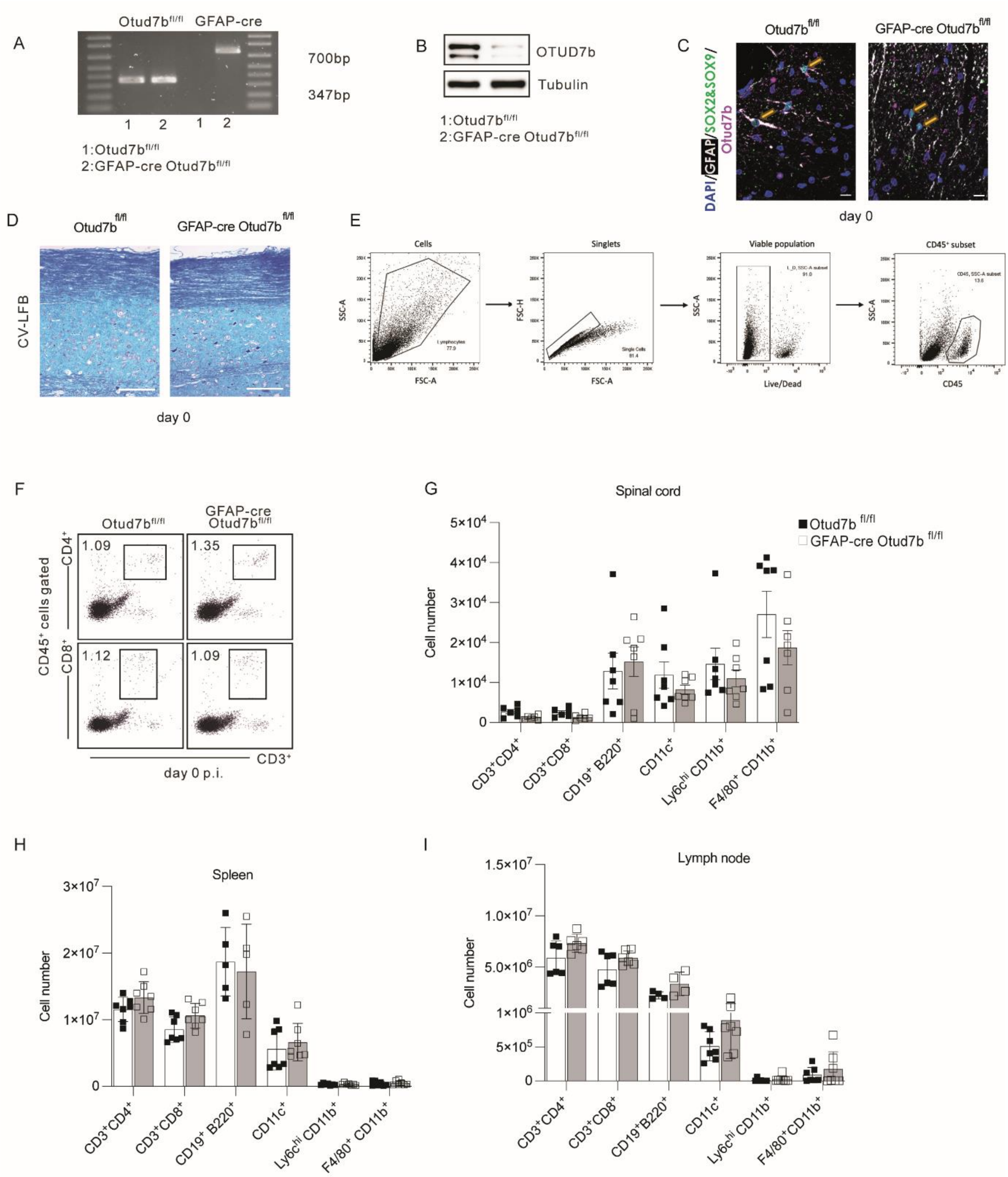

Supplementary Figure 1: OTUD7B in astrocytes does not regulate inflammatory responses under homeostatic conditions

(A-I) Analysis of Otud7b$^{fl/fl}$ and GFAP-cre Otud7b$^{fl/fl}$ mice under homeostatic conditions. (A) Ear biopsies from Otud7b$^{fl/fl}$ and GFAP-cre Otud7b$^{fl/fl}$ mice were genotype for the presence of floxed OTUD7B allele and Cre recombinase under the

GFAP promoter (B) OTUD7B WB of astrocytes isolated from the spinal cords showing deletion of OTUD7b in GFAP-cre Otud7b$^{fl/fl}$ but not in Otud7b$^{fl/fl}$ mice. (C) Immunofluorescence staining showing OTUD7b expression in SOX2/SOX9^{+}GFAP^{+} spinal cord astrocytes (yellow arrow) of unimmunized Otud7b$^{fl/fl}$ but not of GFAP-cre Otud7b$^{fl/fl}$ mice. Scale bars correspond to 10µm. (D) Cresyl-violet luxol-fast blue (CV-LFB) staining in spinal cord of unimmunized Otud7b$^{fl/fl}$ and GFAP-cre Otud7b$^{fl/fl}$ mice showing normal architecture and myelination of spinal cords independent of OTUD7B. Scale bars correspond to 100µm. (E) Gating strategy for CD45^{+} cells. (F) Flow cytometric dots plot showing equal relative numbers of CD3^{+}CD4^{+}CD45^{+} (CD4T cells) and CD3^{+}CD8^{+}CD45^{+} (CD8 T cells) in the spinal cords of Otud7b$^{fl/fl}$ and GFAP-cre Otud7b$^{fl/fl}$ mice. (F-I) Absolute numbers of CD3^{+}CD4^{+}CD45^{+} (CD4 T cells), CD3^{+}CD8^{+}CD45^{+} (CD8T cells), CD19^{+}B220^{+}CD45^{+} (B cells), CD11c^{+} CD45^{+} (dendritic cells), Ly6C^{high} CD11b^{+} CD45^{+} (monocytes), and F4/80^{+} CD11b^{+} CD45^{+} (macrophages) in spinal cord (G), spleen (H), and lymph nodes (I) of non-immunized Otud7b$^{fl/fl}$ mice and GFAP-cre Otud7b$^{fl/fl}$ mice. All bars represent mean values ± SEM (B,D,E).Statistical analyses: two-tailed Student’s t test.

Supplementary figure 2

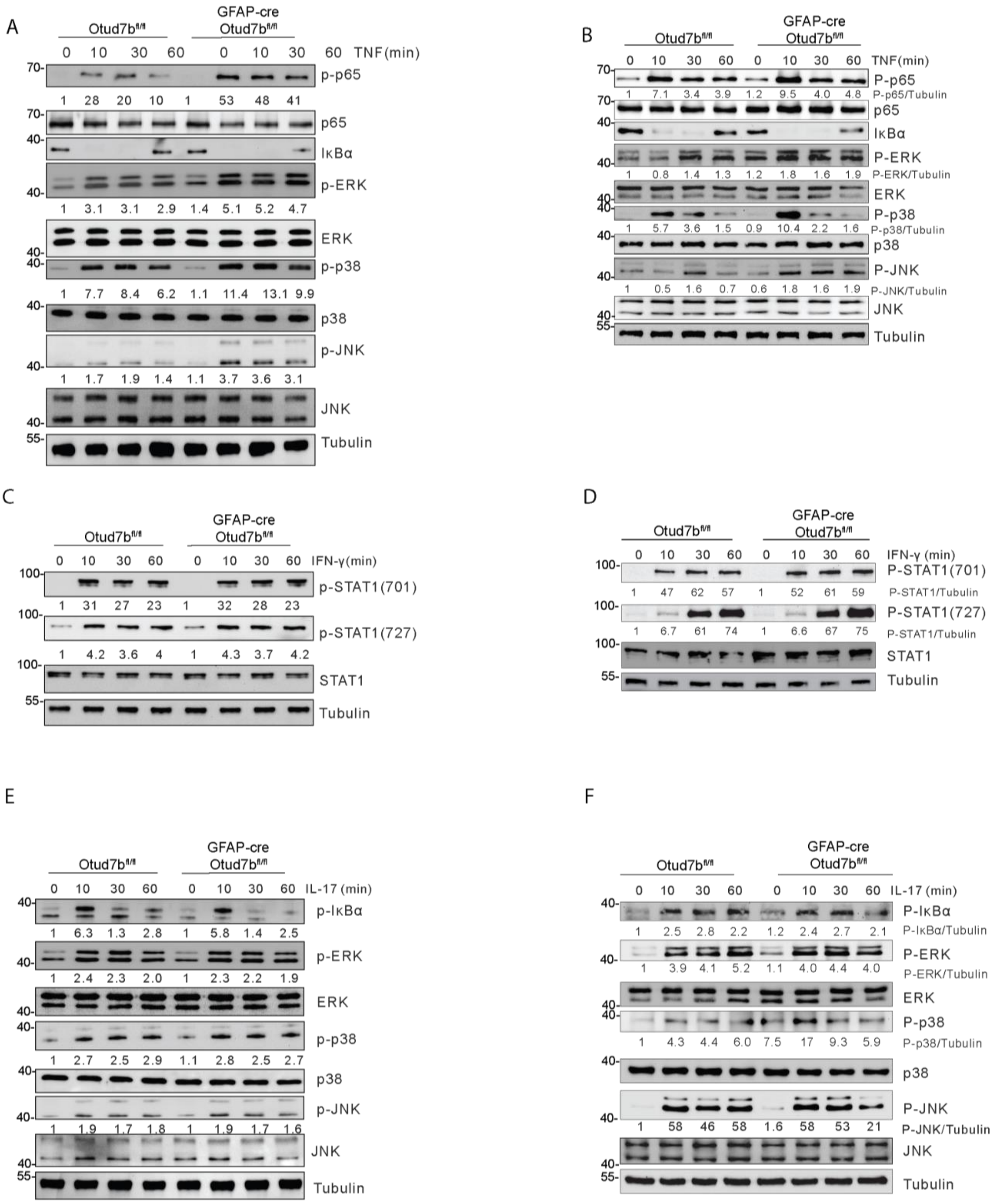

Supplementary Figure 2: OTUD7B impairs TNF mediated pro-inflammatory responses in astrocytes (Repeat WB for Figure 5)

Primary astrocyte cultures were prepared from P0/1 pups of Otud7b$^{fl/fl}$ and GFAP-cre Otud7b$^{fl/fl}$ mice, respectively. Proteins were harvested and analyzed by WB with the indicated antibodies. WB for Figure 5 are shown after (A,B) TNF (20ng/mL), (C,D) IFN-γ (10ng/mL) and (E,F) IL-17 (50ng/mL) stimulation, respectively. Protein expression was normalized to GAPDH and quantified based on WB data

Supplementary figure 3

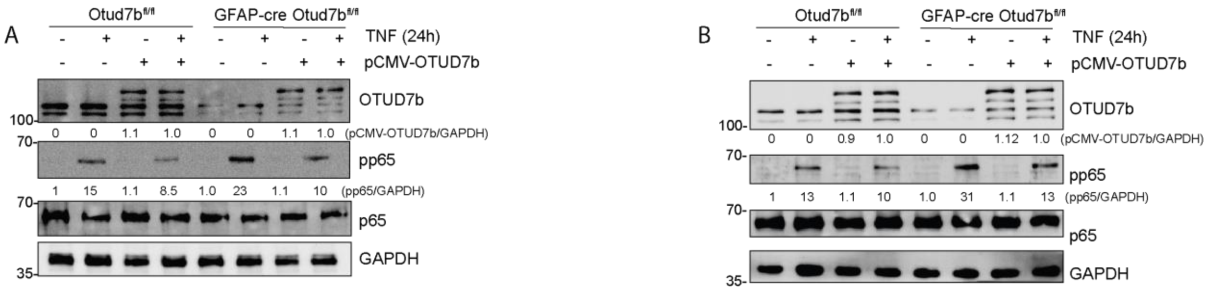

Supplementary Figure 3: Over-expression of OTUD7B impairs TNF mediated pro-inflammatory responses in OTUD7B deficient astrocytes (Repeat WB for Figure 6)

(A,B) Primary astrocyte cultures were prepared from P0/1 pups of Otud7b$^{fl/fl}$ and GFAP-cre Otud7b$^{fl/fl}$ mice, respectively. OTUD7B was overexpressed in both OTUD7B sufficient and deficient astrocytes using the pCMV6-AC-GFP-OTUD7b expression plasmid. Cells were stimulated with TNF (20ng/mL) for 24h and proteins were harvested and analyzed by WB with the indicated antibodies.

Supplementary figure 4

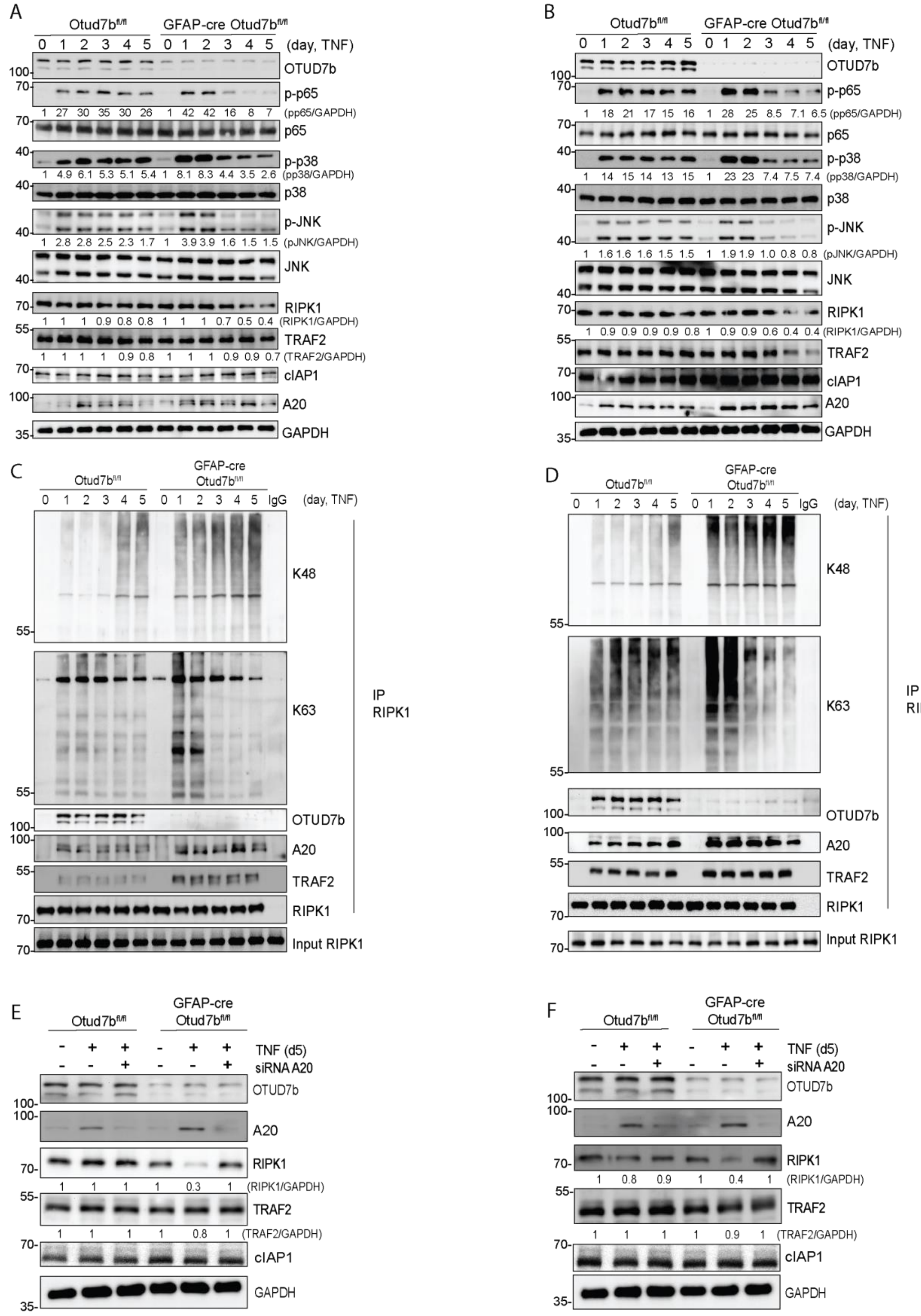

Supplementary Figure 4: Temporal regulation of TNF signaling by OTUD7B (Repeat WB for Figure 7)

OTUD7b-sufficient and -deficient primary astrocytes were stimulated with TNF (20ng/mL). At indicated time points, astrocytes were lysed in RIPA lysis buffer for protein isolation. (A, B) Proteins were immunoblotted with the indicated antibodies. (C, D) Co-immunoprecipitation was performed with anti-RIPK1 antibody followed by immunodetection of the indicated proteins by WB. (E,F) OTUD7B-sufficient and -deficient astrocytes were transfected with non-specific control siRNA or A20 siRNA (5μM) and stimulated with TNF for 5 days. At day 5 post-stimulation, astrocytes were lysed with RIPA buffer and proteins were analyzed by WB using indicated antibodies. Protein expression was normalized to GAPDH and quantified based on WB data

Supplementary figure 5

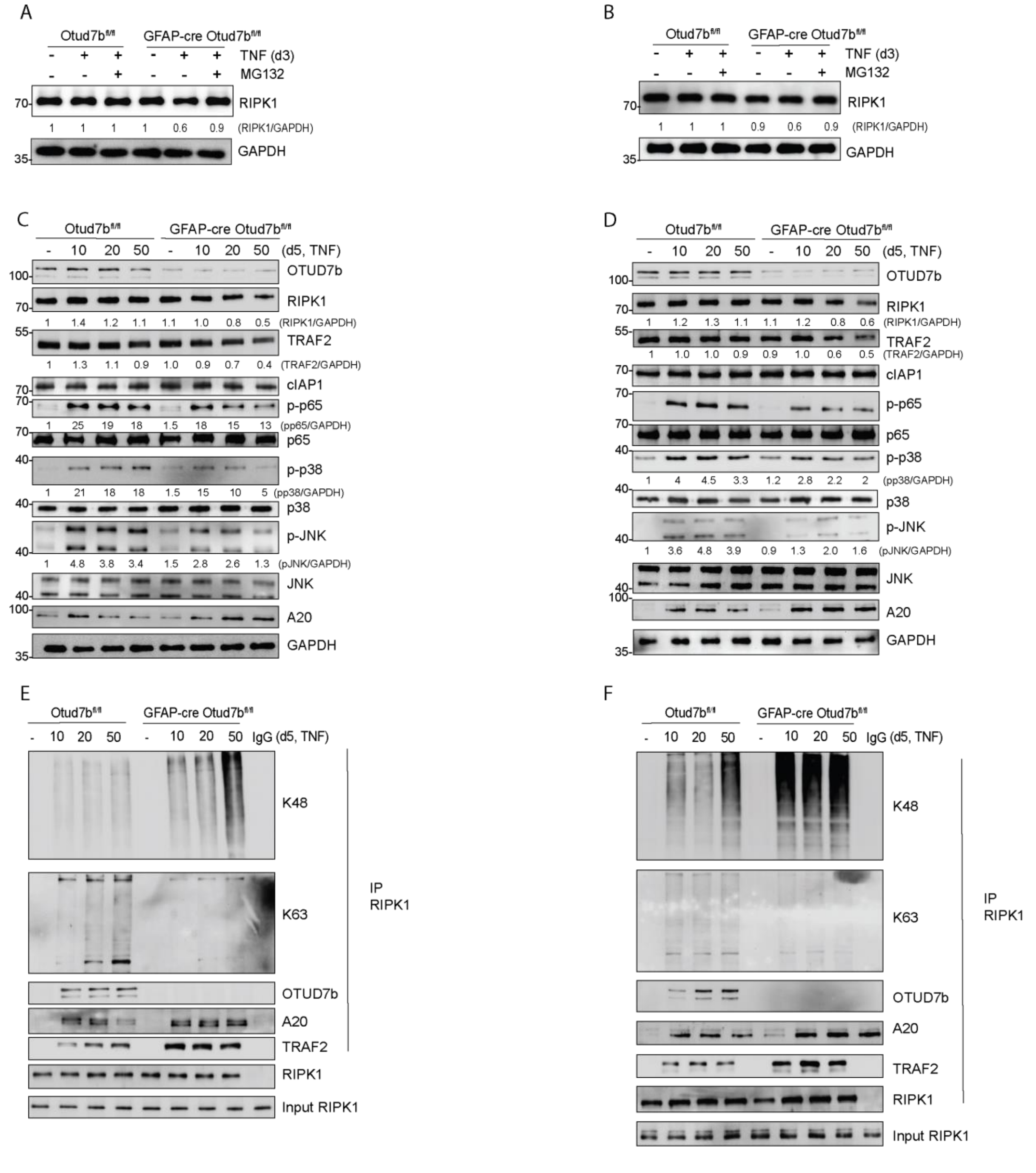

Supplementary Figure 5: Temporal regulation of TNF signaling by OTUD7B (Repeat WB for Figure 7)

(A,B) Astrocytes were treated with TNF for 3 days and MG132 was added on day 3 for 6 hours to inhibit the proteasome. Astrocytes were lysed in RIPA buffer and the protein lysates were analyzed by WB for RIPK1 and GAPDH. (C-F) OTUD7B-sufficient and -

deficient primary astrocytes were stimulated with 10 ng/mL, 20 ng/mL, and 50 ng/mL TNF for 5 days. On day 5 post-stimulation, cells were lysed in RIPA lysis buffer and proteins were isolated for (C, D) analysis of protein expression by WB and (E,F) identification of interacting proteins by co-immunoprecipitation of protein complexes with anti-RIPK1 antibody followed by WB analysis. Protein expression was normalized to GAPDH and quantified based on WB data

Supplementary figure 6

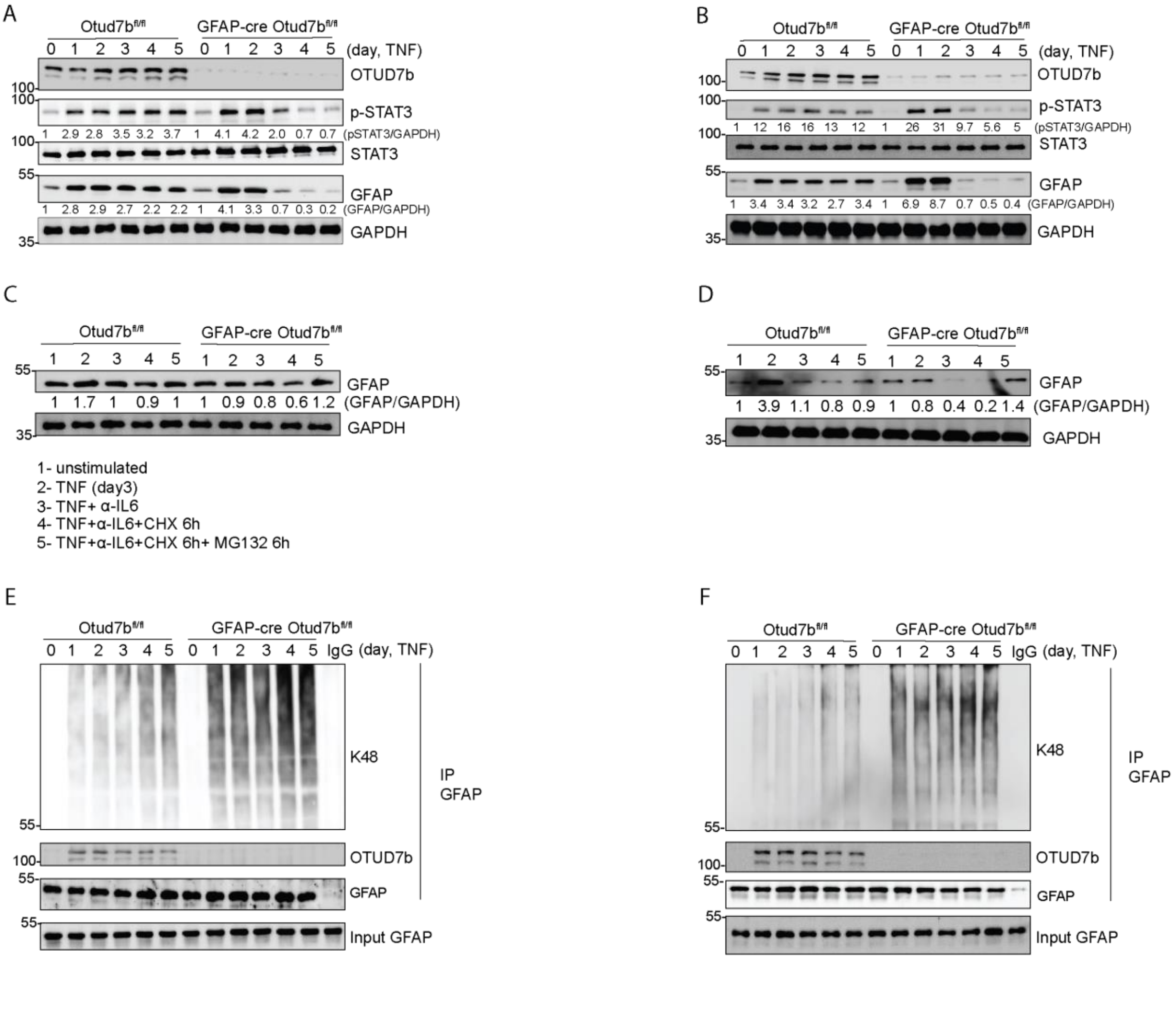

Supplementary Figure 6: OTUD7B prevents K48-ubiquitination of GFAP and its subsequent proteasomal degradation (Repeat WB for Figure 8)

Primary astrocytes were stimulated with 50 ng/mL of TNF and lysed at the indicated time points in RIPA lysis buffer for protein isolation (A-B) WB was performed to analyze the expression levels of OTUD7B, p-STAT3, STAT3, GFAP, and GAPDH. Protein expression was normalized to GAPDH and quantified based on WB data. (C-D) OTUD7B-sufficient and -deficient astrocytes were stimulated with 50 ng/mL of TNF alone, or in combination with anti-IL6 neutralizing antibody for 3 days. On day 3 post-stimulation, 10 μg/mL of CHX and/or 10 μM of MG132 were added to the cells for 6 hours, and proteins were harvested for WB. The expression level of GFAP was analyzed by WB using GAPDH as a loading control. Relative protein expression of GFAP normalized to GAPDH is shown. (E-F) Protein complexes from total protein lysates were immunoprecipitated using anti-GFAP antibody, and precipitates were analyzed for GFAP, OTUD7B, and K48 ubiquitin chains by WB.

Uncropped scans for all blots

Supplementary Figure 1B

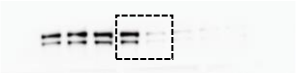

OTUD7b

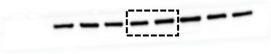

GAPDH

Supplementary figure 2B

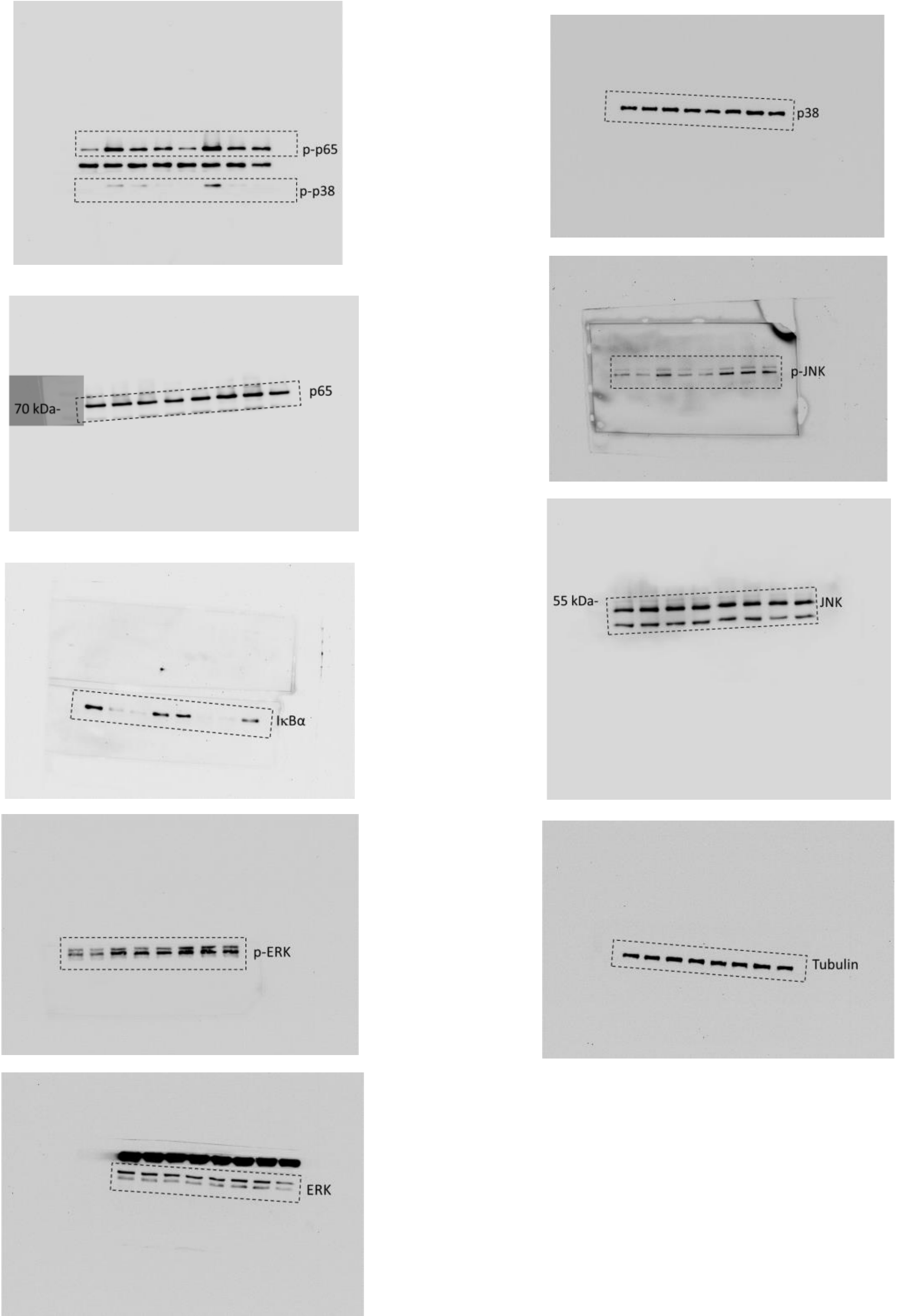

Supplementary figure 2A

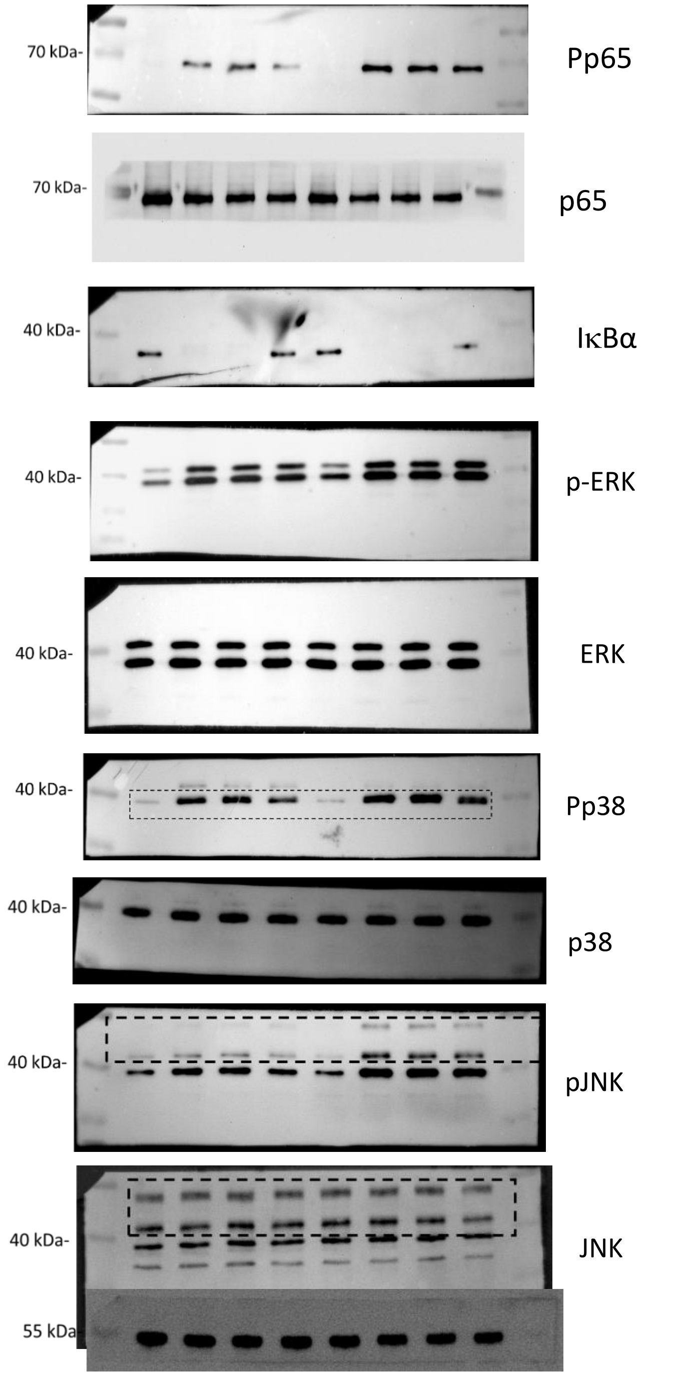

Tubulin

Supplementary figure 2D

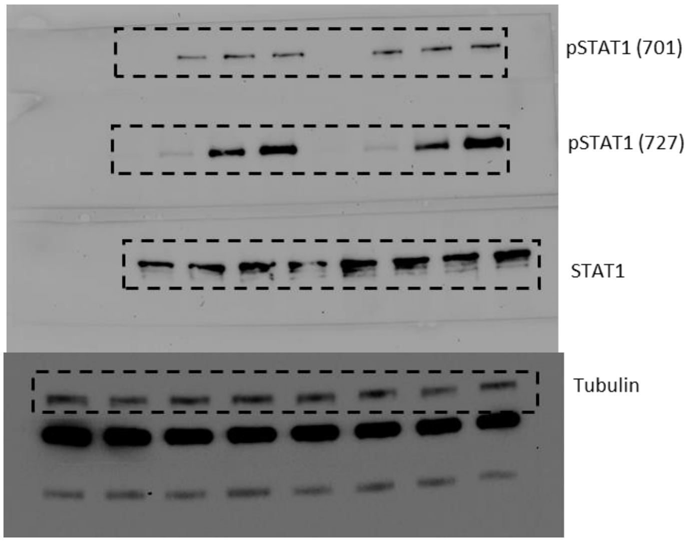

Supplementary figure 2C

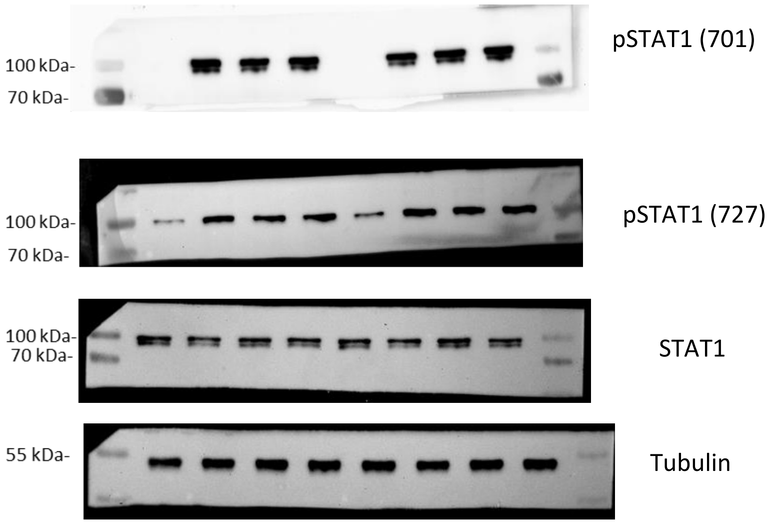

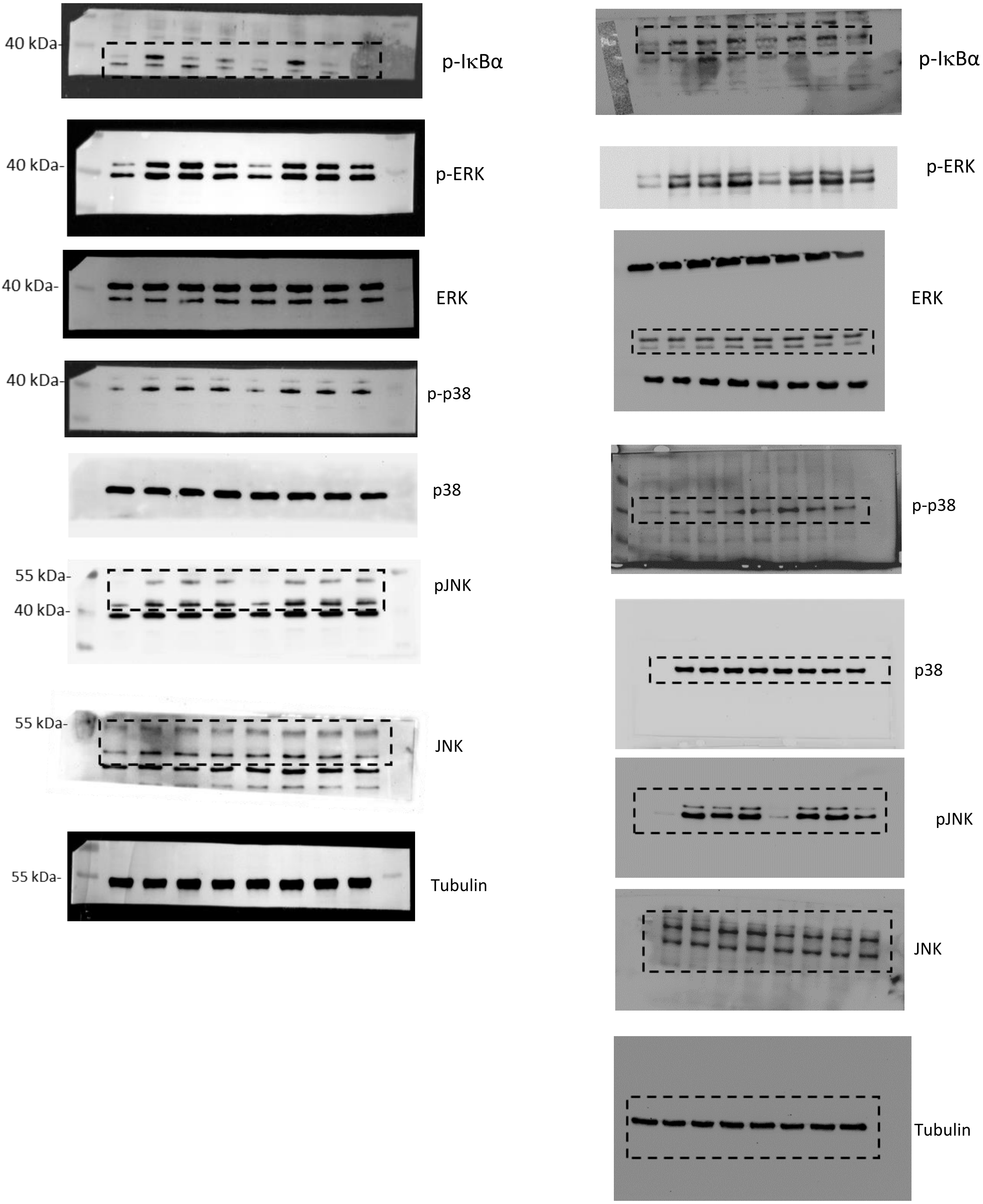
40 kDa-
p-IκBα
40 kDa-
p-ERK
40 kDa-
ERK
40 kDa-
p-p38
p38
55 kDa-
40 kDa-
pJNK
55 kDa-
JNK
55 kDa-
Tubulin
p-IκBα
p-ERK
ERK
p-p38
p38
pJNK
JNK
Tubulin

Supplementary figure 3A

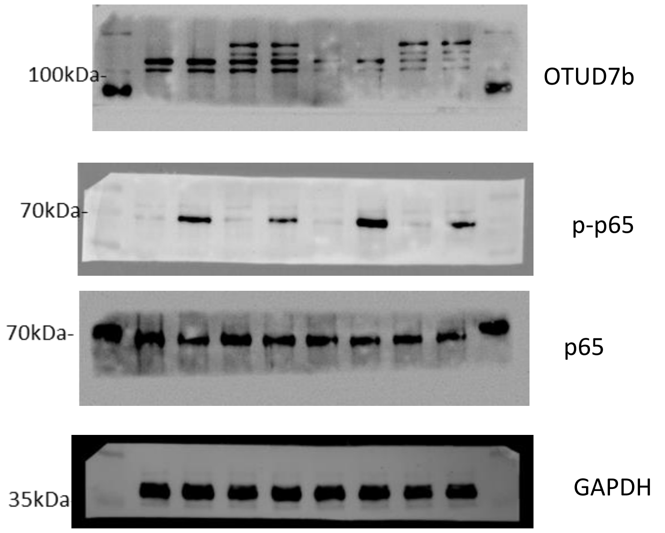

3B

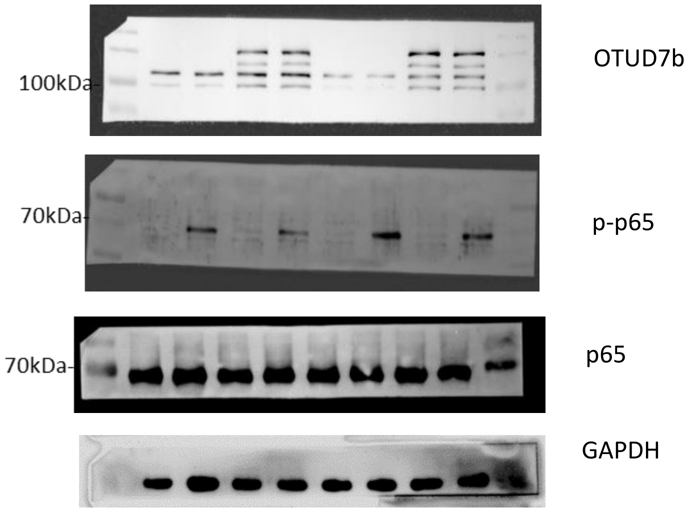

Supplementary figure 4A

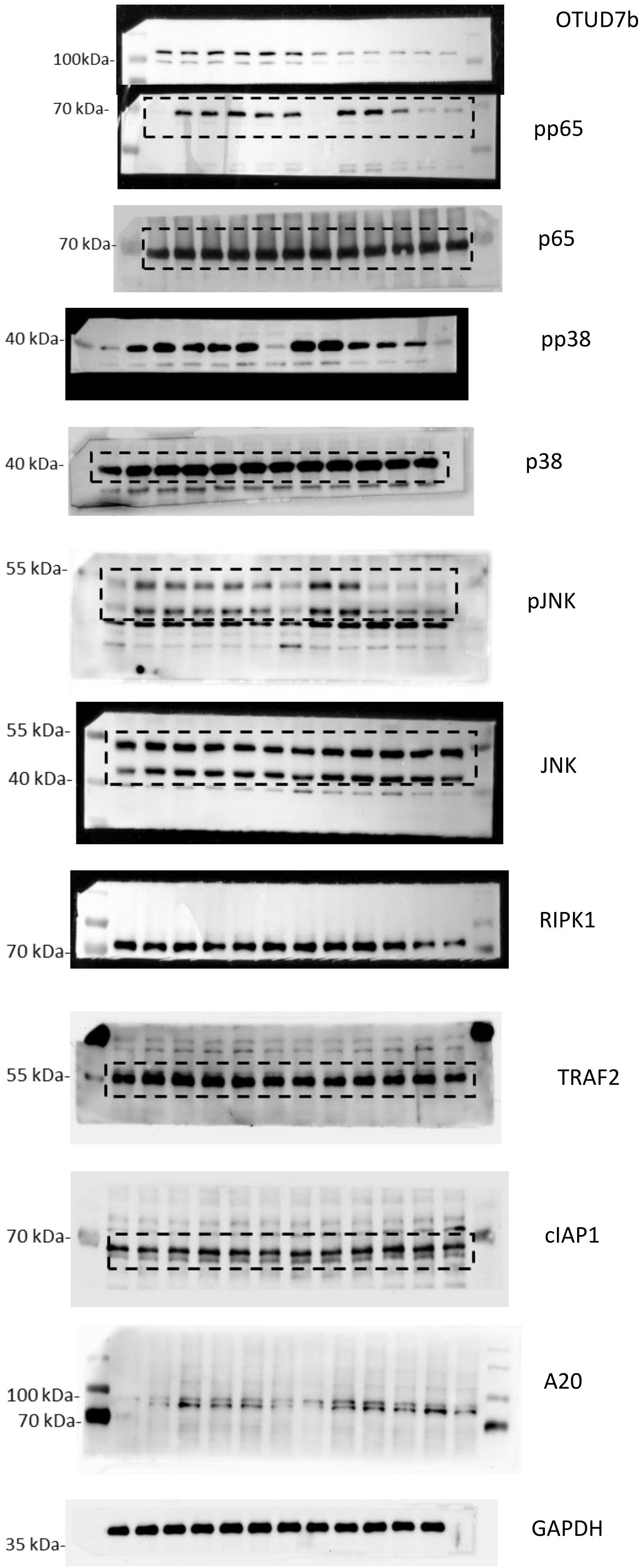

Supplementary figure 4B

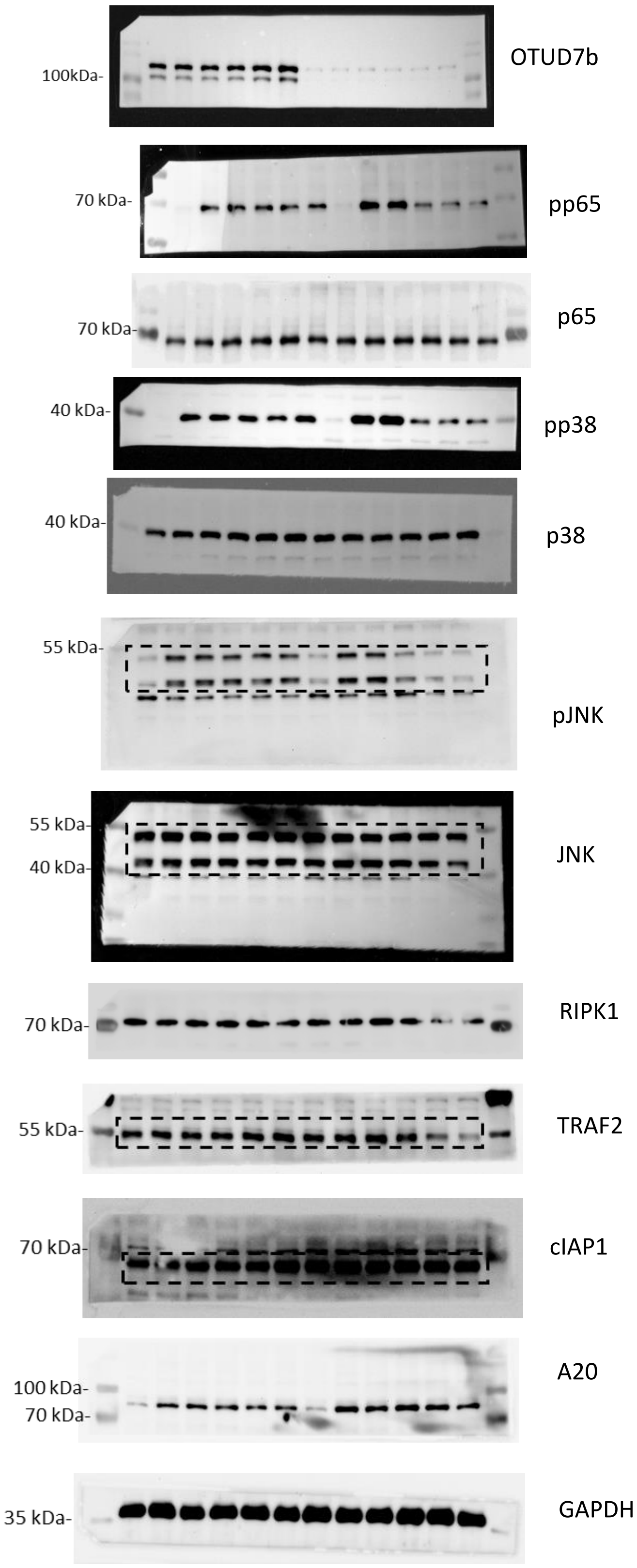

Supplementary figure 4C

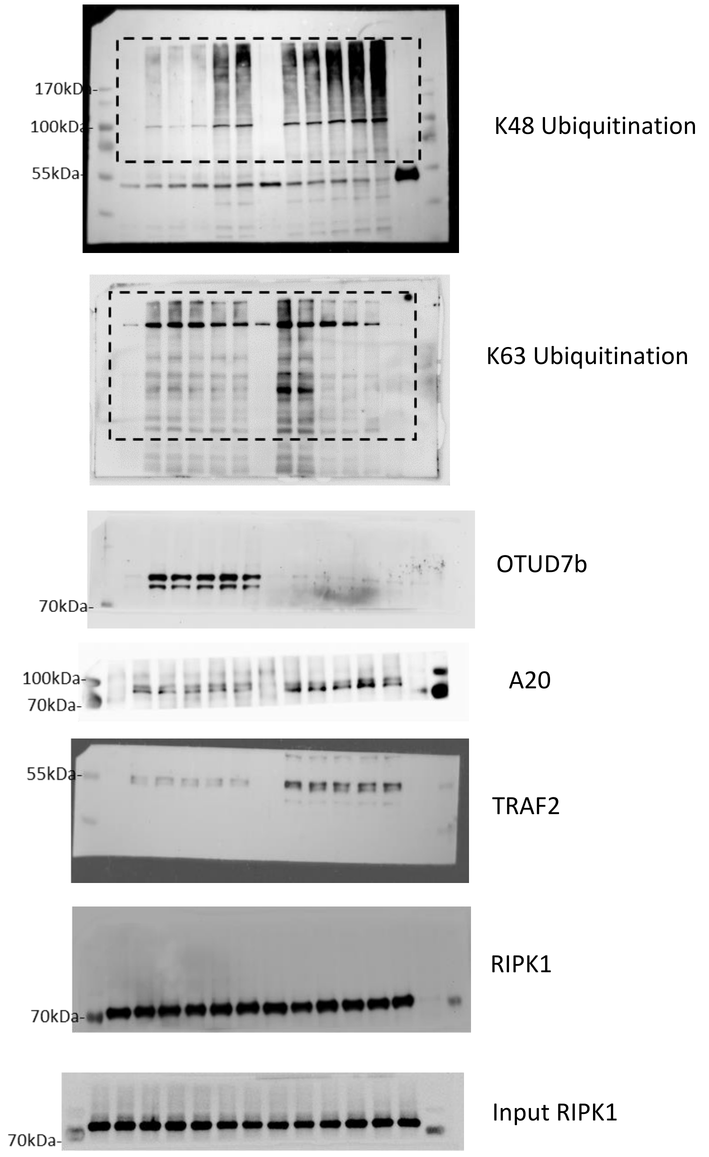

Supplementary figure 4D

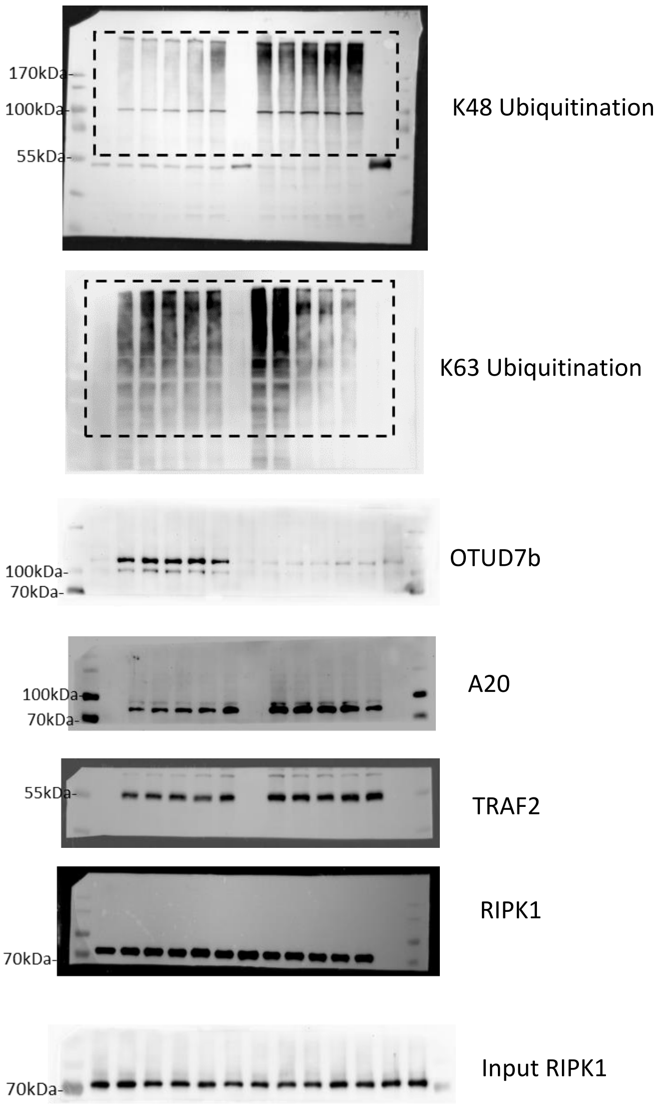

Supplementary figure 4E

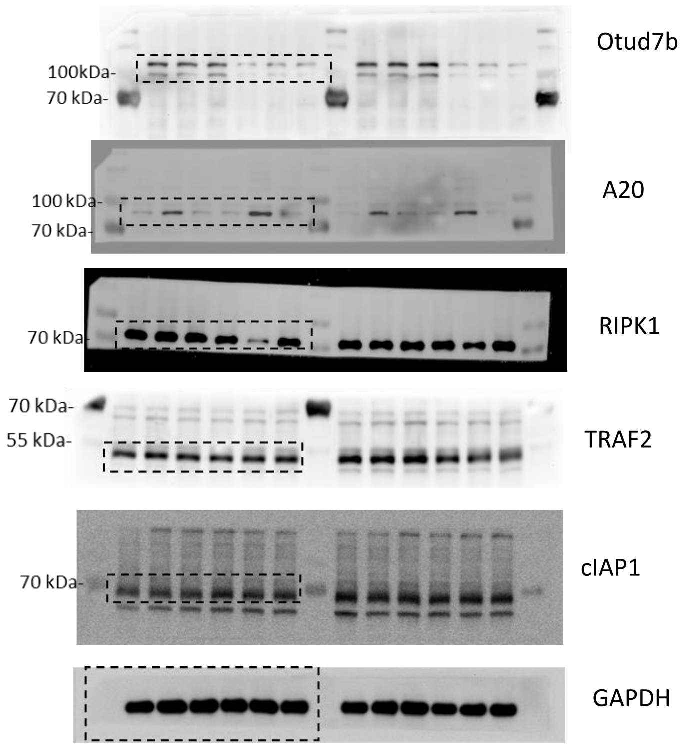

Supplementary figure 4F

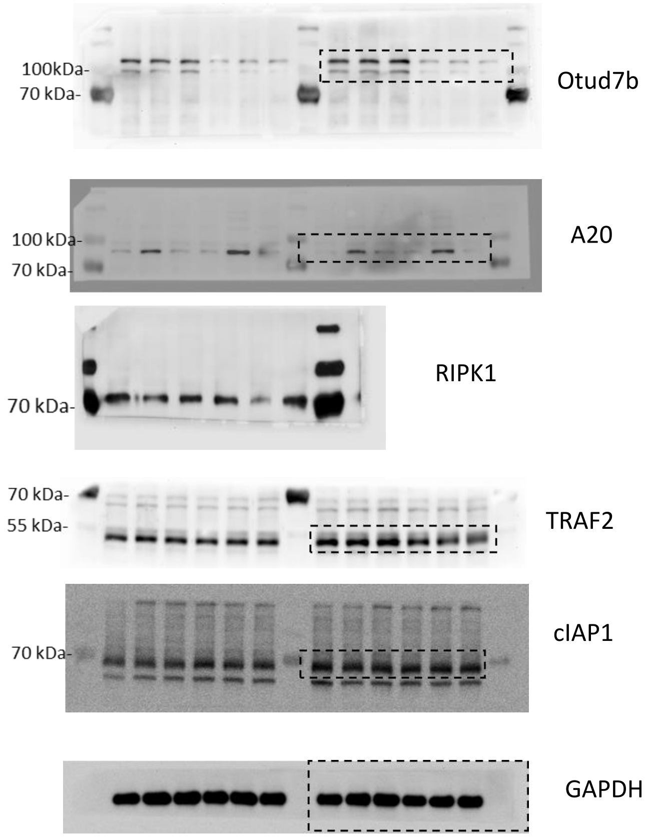

Supplementary figure 5A

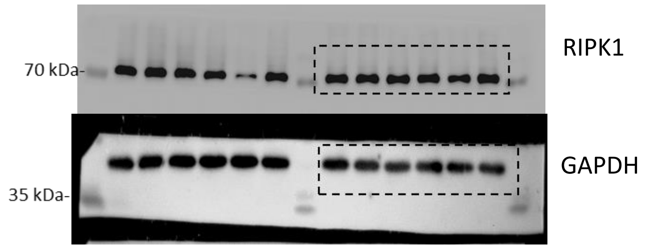

Supplementary figure 5B

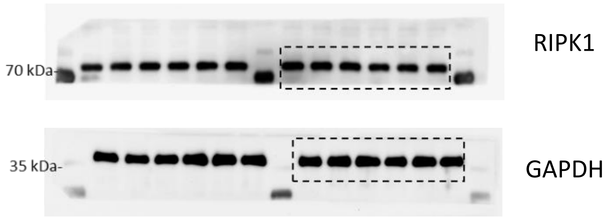

Supplementary figure 5C

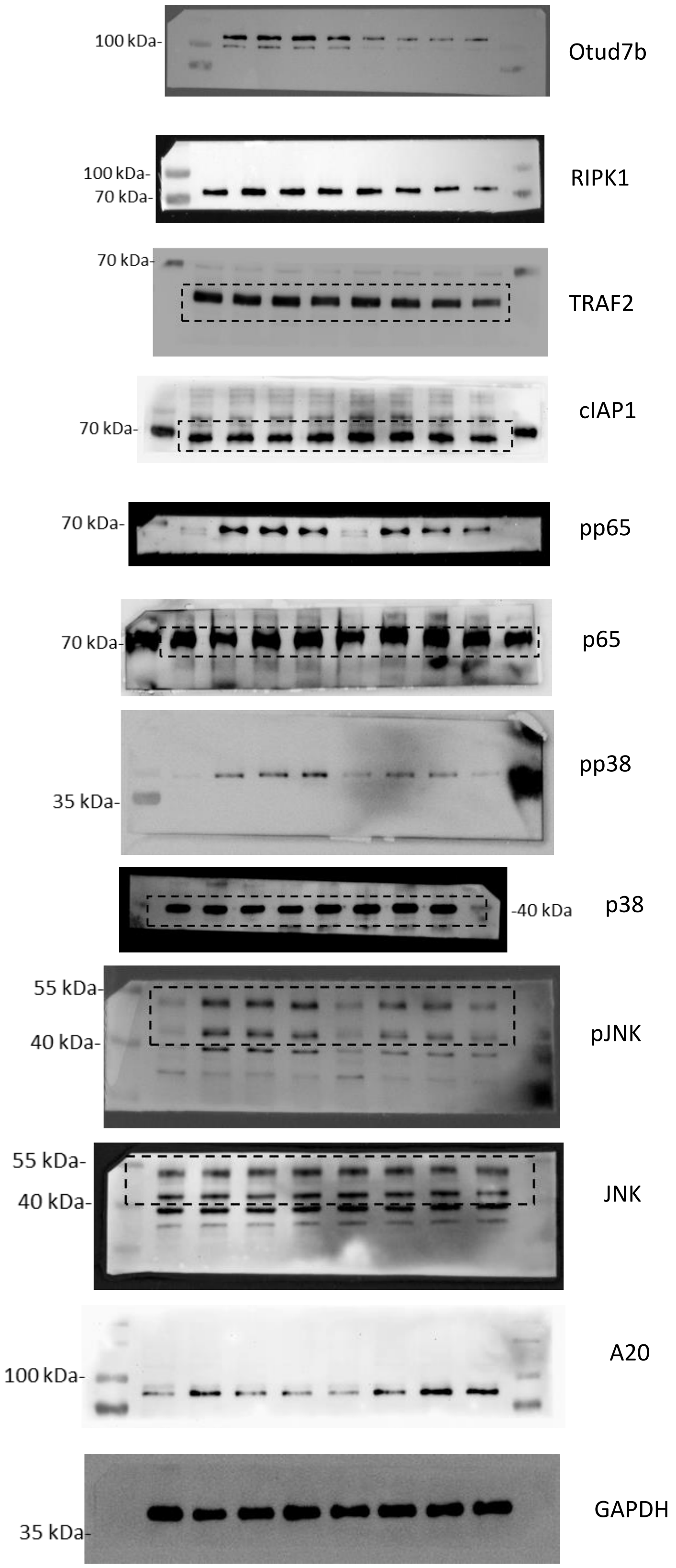

Supplementary figure 5D

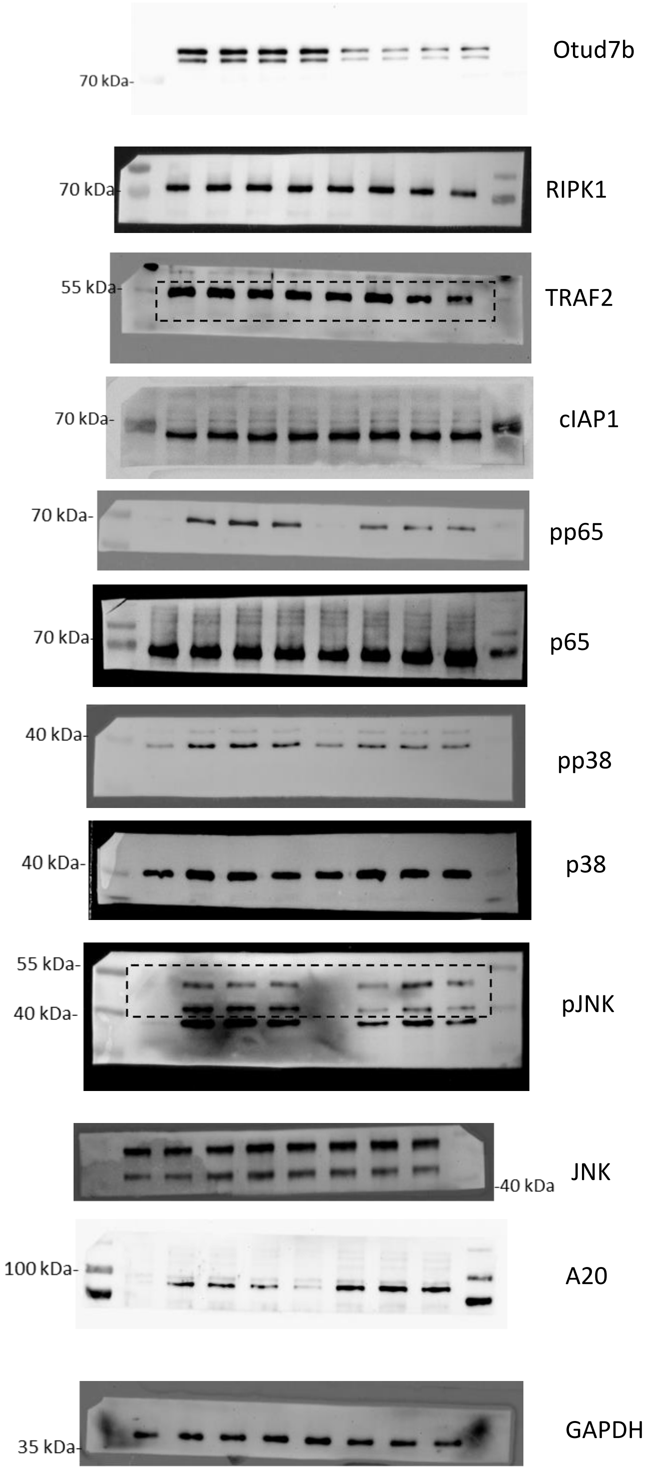

Supplementary figure 5E

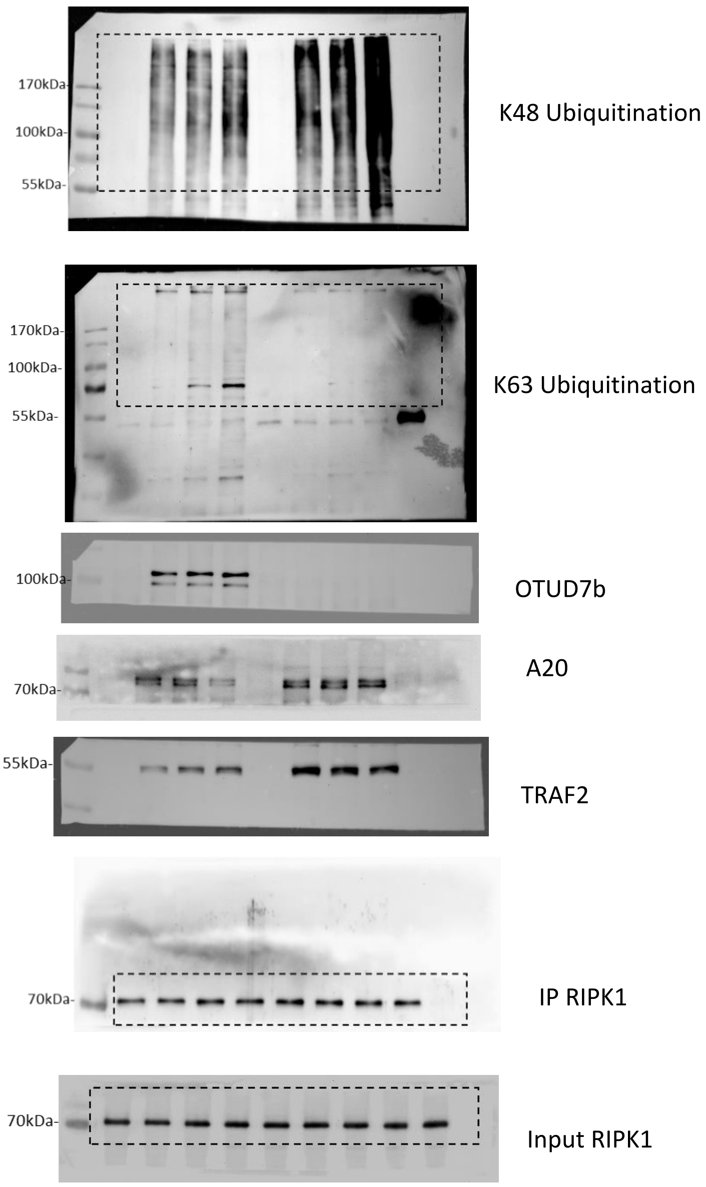

Supplementary figure 5F

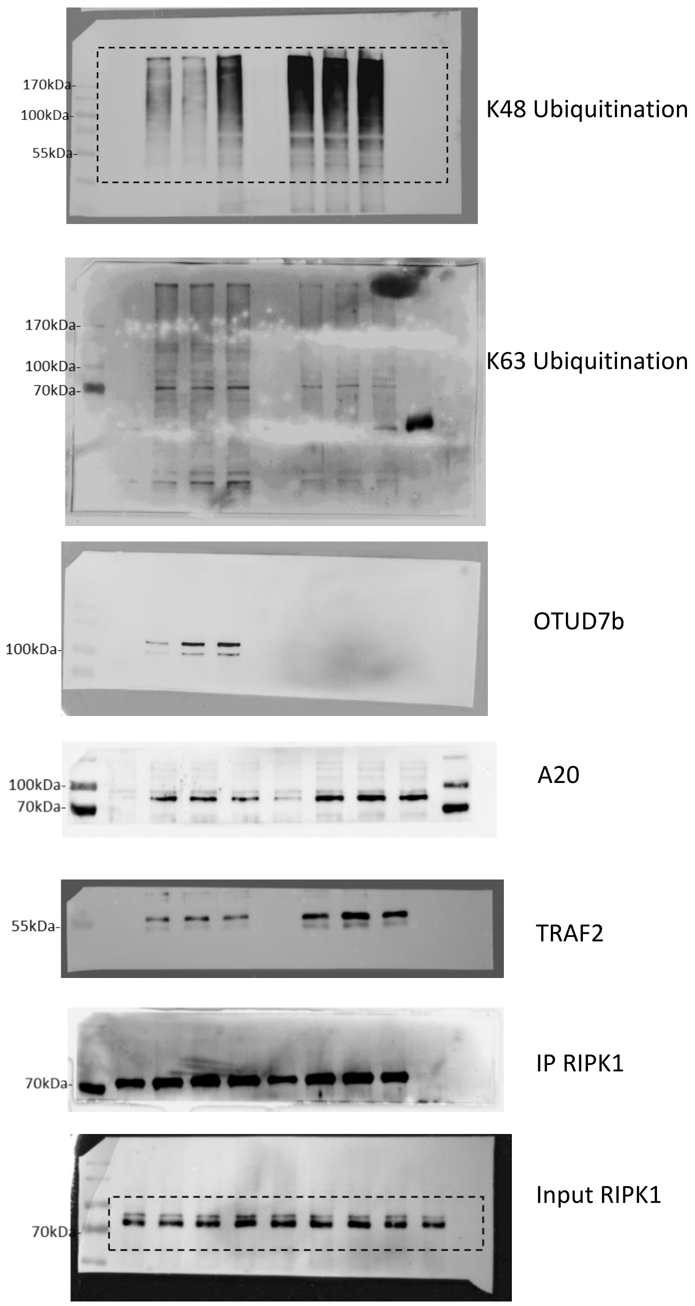

Supplementary figure 6A

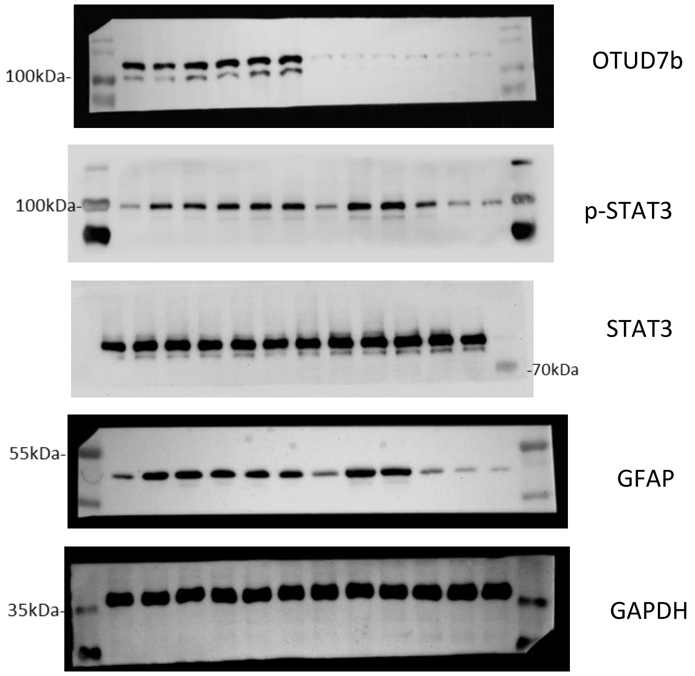

Supplementary figure 6B

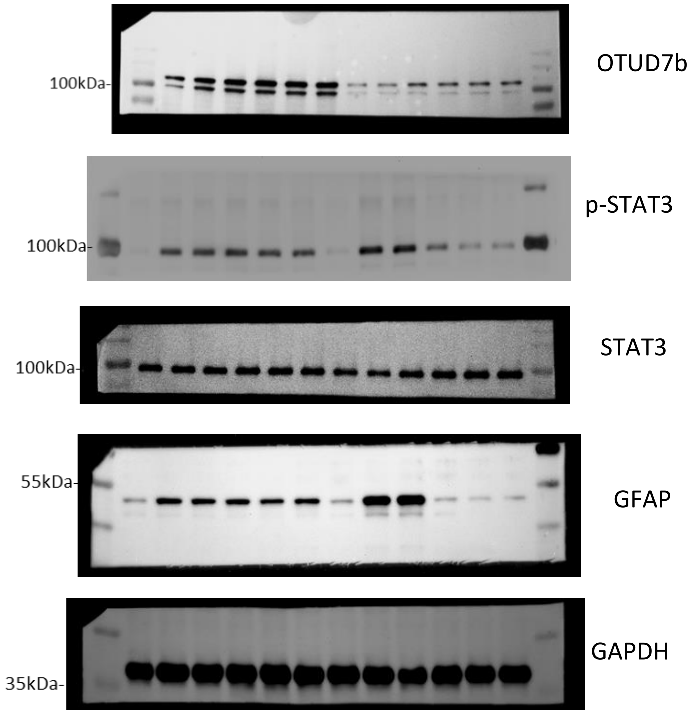

Supplementary figure 6C

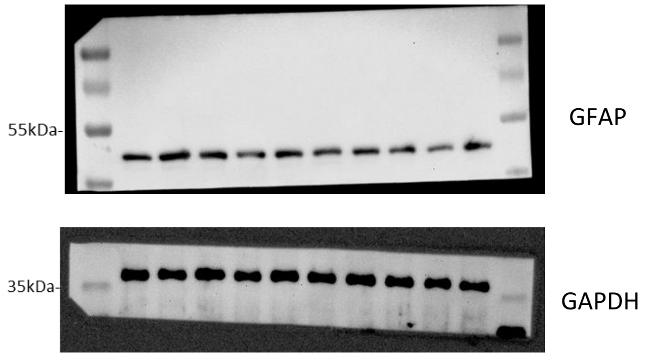

Supplementary figure 6D

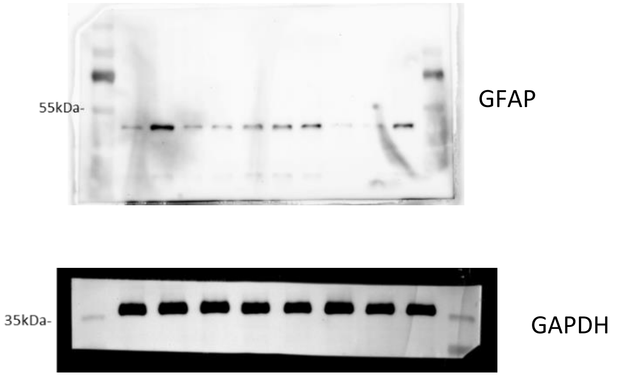

Supplementary figure 6E

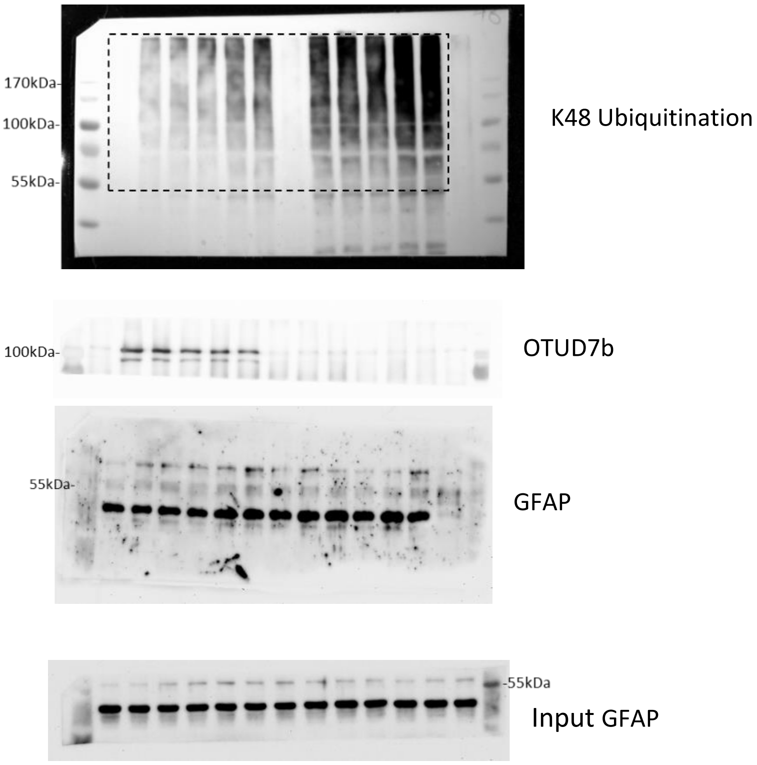

Supplementary figure 6F

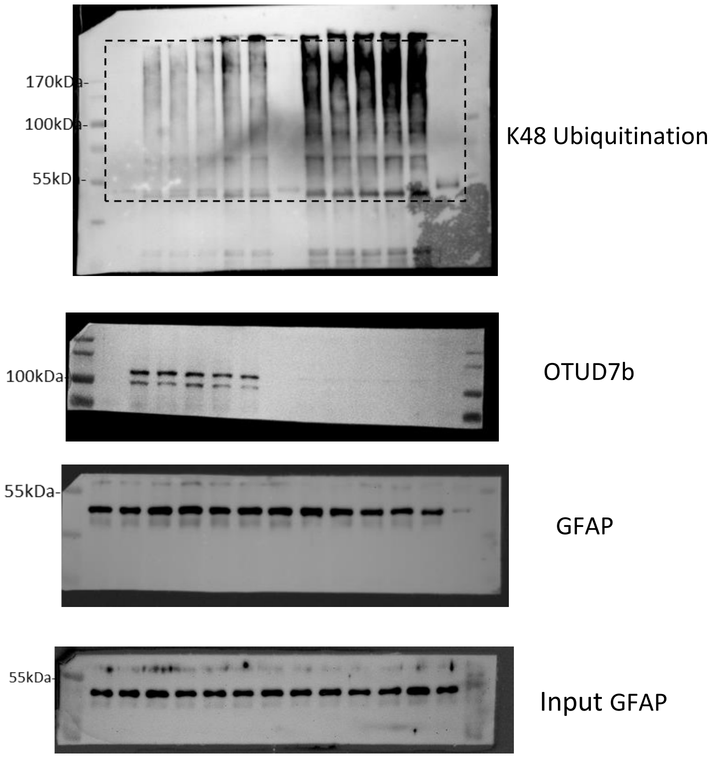

Supplement: Supplementary file 1 — Supplementary Information [file 41467_2025_65093_MOESM1_ESM.pdf]
