# Supplementary material for: Astrocytic-OTUD7B ameliorates murine experimental autoimmune encephalomyelitis by stabilizing glial fibrillary acidic protein and preventing inflammation
Source: Nat Commun. 2025 Oct 20;16:9279. doi: 10.1038/s41467-025-65093-4 (PMC12537900; doi:10.1038/s41467-025-65093-4)
Supplement: Supplementary file 2 — Description of additional supplementary file [file 41467_2025_65093_MOESM2_ESM.docx]

Supplementary dataset 1:

List of pre-designed mouse brain panel with 247 genes in combination with a custom panel including additional 50 genes by Xenium used for spatial transcriptomics analysis.

Supplementary dataset 2:

List of reagents used in this study
