# Supplementary material for: Astrocytic-OTUD7B ameliorates murine experimental autoimmune encephalomyelitis by stabilizing glial fibrillary acidic protein and preventing inflammation
Source: Nat Commun. 2025 Oct 20;16:9279. doi: 10.1038/s41467-025-65093-4 (PMC12537900; doi:10.1038/s41467-025-65093-4)
Supplement: Supplementary file 4 — Supplementary Dataset 2 [file 41467_2025_65093_MOESM4_ESM.docx]

| **Reagent or Resource** | **Source** | **Identifier** | **Source** | **Dilution** |
| --- | --- | --- | --- | --- |
| **Antibodies for Western blotting** | | |  |  |
| α-OTUD7b | Proteintech | 16605-1-AP | Rabbit | 1:1000 |
| α-GFAP | Proteintech | 16823-1-AP | Rabbit | 1:5000 |
| α- β Tubulin | Proteintech | 10068-1-AP | Rabbit | 1:5000 |
| α-GAPDH | Cell Signaling Technology | 2118 | Rabbit | 1:5000 |
| α-phospho STAT1(Tyr 701) | Cell Signaling Technology | 9167 | Rabbit | 1:5000 |
| α-phospho STAT1(Ser 727) | Cell Signaling Technology | 9177 | Rabbit | 1:1000 |
| α-STAT1 | Cell Signaling Technology | 9172 | Rabbit | 1:5000 |
| α-phospho-p65 | Cell Signaling Technology | 3031 | Rabbit | 1:1000 |
| α-IκBα | Cell Signaling Technology | 4812 | Rabbit | 1:1000 |
| α-p65 | Cell Signaling Technology | 8242 | Rabbit | 1:1000 |
| α-phospho-p38 MAPK | Cell Signaling Technology | 9215 | Rabbit | 1:1000 |
| α-p38 MAPK | Cell Signaling Technology | 9212 | Rabbit | 1:1000 |
| α-phospho-p44/42 MAPK | Cell Signaling Technology | 9101 | Rabbit | 1:1000 |
| α-p44/42 MAPK | Cell Signaling Technology | 9102 | Rabbit | 1:1000 |
| α-TRAF2 | Cell Signaling Technology | 4724 | Rabbit | 1:1000 |
| α-RIPK1 | Cell Signaling Technology | 3493 | Rabbit | 1:1000 |
| α-phospho JNK | Cell Signaling Technology | 4668 | Rabbit | 1:1000 |
| α-JNK | Cell Signaling Technology | 9252 | Rabbit | 1:1000 |
| α-TNFAIP3/A20 | Cell Signaling Technology | 5630 | Rabbit | 1:1000 |
| α-phospho STAT3 | Cell Signaling Technology | 9131 | Rabbit | 1:1000 |
| α-STAT3 | Cell Signaling Technology | 9139 | Mouse | 1:5000 |
| α-cIAP1 | Abcam | ab154525 | Rabbit | 1:1000 |
| α-ubiquitin Lys48-specific | Merck Millipore | 05-1307 | Rabbit | 1:1000 |
| α-ubiquitin Lys63-specific | Merck Millipore | 05-1308 | Rabbit | 1:1000 |
| α-ubiquitin Lys11-specific | Merck Millipore | MABS-107-I | Rabbit | - |
| Rabbit α-mouse IgG/HRP | Dako | P0161 | Rabbit | - |
| Swine α-rabbit IgG/HRP | Dako | P0399 | Swine | - |
| Mouse α-rabbit IgG, light chain specific | Jackson ImmunoResearch | AB_2339149 | Mouse | - |
| Goat α-mouse IgG, light chain specific | Jackson ImmunoResearch | AB_2338512 | Goat | - |
| **Flourochrome conjugated antibodies for flow cytometry** | | |  |  |
| α-CD3e-APC-Cy7 | BioLegend | 100204, 17A2 | Rat | 1:100 |
| α-CD3e-PE | eBioscience | 12-0033-82, REA975 | Hamster | 1:100 |
| α-CD4-BV421 | BioLegend | 100443, GK1.5 | Rat | 1:100 |
| α- CD8α-APC | eBioscience | 17-0081-82, 53-6.7 | Rat | 1:100 |
| α-CD8α-FITC | eBioscience | 11-0081-85, 53-6.7 | Rat | 1:100 |
| α-CD45-PerCP | BioLegend | 103130, 30-F11 | Rat | 1:100 |
| α-CD11b-PE-Cy7 | BioLegend | 101216, M1/70 | Rat | 1:100 |
| α-F4/80-BV421 | BioLegend | 123132, BM8 | Rat | 1:100 |
| α-Ly6C-APC | eBioscience | 17-5932-82, HK1.4 |  | 1:100 |
| α-Ly6G-PE | BioLegend | 127608, 1A8 | Rat | 1:100 |
| α-CD19-PE | eBioscience | 12-0193-82, 1D3 | Rat | 1:100 |
| α-CD45R/B220-BV421 | BD Bioscience | 562922, RA3-6B2 | Rat | 1:100 |
| α-CD11c-PE | eBioscience | 12-0114-83, N418 | Hamster | 1:100 |
| α-ACSA-2-PE | eBioscience | 130-123-284, IH3-18A3 | Rat | 1:100 |
| α-IFN- γ | eBioscience | 12-7311-82, XMG1.2 | Rat | 1:100 |
| α-IL-17 | BioLegend | 506904, TC11-18H10.1 | Rat | 1:100 |
| α-GM-CSF | BioLegend | 505406, MP1-22E9 | Rat | 1:100 |
| **Primary antibodies for Immunohistochemistry and histology** | | |  |  |
| α-GFAP | Agilent | AB_2811722 | Rabbit | 1:1000 |
| α-Sox2 | Abcam | AB_10710406 | Mouse | 1:500 |
| α-Sox9 | RnD Systems | AB_2194160 | Mouse | 1:500 |
| α-Iba-1 | Abcam | AB_283346 | Rat | 1:800 |
| α-GFAP | Dako | GA52461-2 | Rabbit | 1:1000 |
| DAPI | Thermo Fischer Scientific | D1306 |  | - |
| Alexa488-conjugated donkey anti-goat | Thermo Fischer Scientific | AB_2534102 | Donkey | - |
| Alexa488-conjugated donkey anti-mouse | Thermo Fischer Scientific | AB_141607 | Donkey | - |
| Cy3-conjugated donkey anti-rabbit | Jackson | AB_2340607 | Donkey | - |
| Cy5-conjugated donkey anti-rabbit | Jackson | AB_2340607 | Donkey | - |
|  |  |  |  |  |
|  |  |  |  |  |
| **Reagent or Resource** | **Source** | **Identifier** |  |  |
| *Cxcl-1* | Thermo Fisher Scientific | Mm00433859 |  |  |
| *Cxcl-10* | Thermo Fisher Scientific | Mm00445235 |  |  |
| *Cxcl-11* | Thermo Fisher Scientific | Mm00444662 |  |  |
| *Ccl-2* | Thermo Fisher Scientific | Mm00441242 |  |  |
| *Ccl-20* | Thermo Fisher Scientific | Mm01268754 |  |  |
| *Nos-2* | Thermo Fisher Scientific | Mm00440485 |  |  |
| *Gfap* | Thermo Fisher Scientific | Mm01253033 |  |  |
| *Otud7b* | Thermo Fisher Scientific | Mm01256852 |  |  |
| *Il-17* | Thermo Fisher Scientific | Mm00439619 |  |  |
| *Hprt* | Thermo Fisher Scientific | Mm01545399 |  |  |
| *Ifn-*γ | Thermo Fisher Scientific | Mm00801778 |  |  |
| *Il-6* | Thermo Fisher Scientific | Mm00446190 |  |  |
| *Tnf* | Thermo Fisher Scientific | Mm00443258 |  |  |
| *Csf-2* | Thermo Fisher Scientific | Mm01290062 |  |  |
| **Deposited data** | | |  |  |
| Bulk RNA-Seq data | GSE286263 |  |  |  |
| Spatial transcriptomics data | GSE286422 |  |  |  |
| **Experimental models: Organisms/strains** | | |  |  |
| Mouse: C57BL/6 Otud7b^fl/fl^ | Harit et al | PMID: 37516734 |  |  |
| Mouse: C57BL/6 GFAP-Cre Otud7b^fl/fl^ | This paper | N/A |  |  |
| **Recombinant proteins, peptides and Commercial kits** | | |  |  |
| murine TNF | Peprotech | 315-01A |  |  |
| murine IFNγ | Peprotech | 315-05 |  |  |
| murine IL-17 | Peprotech | 210-17 |  |  |
| MOG_35-55_ peptide | JPT | 163913-87-9 |  |  |
| *Mycobacterium tuberculosis* H37Ra | Fisher Scientific | DF3114-33-8 |  |  |
| Freund’s adjuvant complete | Merck Millipore | AR001 |  |  |
| Pertussis toxin | Sigma-Aldrich | 516560 |  |  |
| RNeasy Mini kit | Qiagen | 74104 |  |  |
| Neurocult Enzymatic dissociation kit | Stemcell Technologies | 05715 |  |  |
| SuperScript Reverse Transcriptase kit | Thermo Fisher Scientific | 18080093 |  |  |
| NEBNext® UltraTM II Directional RNA Library Prep Kit | New England Biolabs | E7760 |  |  |
| H&E Staining Kit (Hematoxylin and Eosin) | Abcam | ab245880 |  |  |
| Pierce ECL Plus Western Blotting Substrate | Thermo Fischer Scientific | 32132 |  |  |
| BCA assay kit | Thermo Fischer Scientific | 23225 |  |  |
| Intracellular Fixation/ Permeabilization kit | eBioscience | 88-8824-00 |  |  |
| PageRuler Prestained protein ladder | Thermo Fisher Scientific | 26616 |  |  |
| KAPA PROBE FAST qPCR master mix | KAPA Biosystems | KK4701 |  |  |
| anti-ACSA-2 microbead kit | Miltenyi Biotech | 130-097-679 |  |  |
| Xenium slides & sample prep reagents kit | 10x genomics | PN-1000460 |  |  |
| Post-Xenium analyzer H&E staining user guide | 10x genomics | CG000613 |  |  |
| **Chemicals and reagents** | | |  |  |
| RIPA lysis buffer | Cell Signalling Technologies | 9806 |  |  |
| Phenylmethylsulfonyl fluoride (PSMF) | Cell Signalling Technologies | 8553 |  |  |
| Protease Inhibitor Cocktail | Sigma Aldrich | PPC2020 |  |  |
| GammaBind G Sepharose Beads | Cytiva | 17061801 |  |  |
| Lane marker reducing sample buffer | Thermo Fisher Scientific | 39000 |  |  |
| Dulbecco's Modified Eagle Medium (DMEM) | Gibco | 41965-039 |  |  |
| RPMI 1640 medium | Gibco | A1049101 |  |  |
| Trypsin-EDTA | Gibco | 25200-056 |  |  |
| Non-essential amino acids | Gibco | 11140050 |  |  |
| L-glutamine | Gibco | 25030081 |  |  |
| Brefeldin A solution 1000X | BioLegend | 420601 |  |  |
| Percoll | Cytiva | 17-0891-02 |  |  |
| Ionomycin | Sigma Aldrich | I3909 |  |  |
| GolgiPlug | BD Bioscience | 555029 |  |  |
| Phorbol 12-myristate 13-acetate | Sigma Aldrich | P1585 |  |  |
| PBS | Sigma Aldrich | D8537 |  |  |
| Trypsin | Gibco | 25200-056 |  |  |
| Cycloheximide | Sigma-Aldrich | 239765 |  |  |
| MG132 | Sigma-Aldrich | M7449 |  |  |
| Cresyl violet | Carl Roth | 7651.3 |  |  |
| Luxol-fast blue | Sigma Aldrich | L0294 |  |  |
| Hematoxylin and eosin | Carl Roth | 9194.2 |  |  |
| 4% Paraformaldehyde | Thermo Fischer Scientific | J61899-AK |  |  |
| 3,3’ diaminobenzidine | Merck | D12384 |  |  |
| Pierce^TM^ ECL plus western blotting substrate | Thermo Fischer Scientific | 32132 |  |  |
| Xenium nuclei staining buffer | 10x Genomics | 2000762 |  |  |
| A20 siRNA | Dharmacon | L-058907-02-0010 |  |  |
| 70um cell strainer | Corning | 352350 |  |  |
| Fixable viability dye eFluor780 | eBioscience | 65-0865-14 |  |  |
| **Instruments and Softwares** | | |  |  |
| LightCycler 480 | Roche | N/A |  |  |
| Cytek Northern light flow cytometer | Cytek | N/A |  |  |
| NovaSeq 6000 sequencer | Illumina | N/A |  |  |
| Xenium analyzer | 10x genomics | N/A |  |  |
| Axiocam 503 | Zeiss | N/A |  |  |
| ApoTome.2 | Zeiss | N/A |  |  |
| Zeiss LSM 780 | Zeiss | N/A |  |  |
| Intas Chemo Cam luminescent image analysis system | INTAS science imaging instruments | N/A |  |  |
| FlowJo V10 | FlowJo | https://www.flowjo.com |  |  |
| GraphPad Prism 10 | Graphpad Software | https://www.graphpad.com |  |  |
| LabImage 1D software | Kapelan Bio-Imaging | N/A |  |  |
| DAVID Functional Annotation Bioinformatics Microarray Analysis |  | https://david.ncifcrf.gov/ |  |  |
| Apotome acquision Zen2.6 pro | Zeiss | N/A |  |  |
