# Supplementary material for: Astrocytic-OTUD7B ameliorates murine experimental autoimmune encephalomyelitis by stabilizing glial fibrillary acidic protein and preventing inflammation
Source: Nat Commun. 2025 Oct 20;16:9279. doi: 10.1038/s41467-025-65093-4 (PMC12537900; doi:10.1038/s41467-025-65093-4)
Supplement: Supplementary file 5 — Reporting Summary [file 41467_2025_65093_MOESM5_ESM.pdf]

nature portfolio

Corresponding author(s): Schlüter D

Last updated by author(s): Sep 19, 2025

Reporting Summary

Nature Portfolio wishes to improve the reproducibility of the work that we publish. This form provides structure for consistency and transparency in reporting. For further information on Nature Portfolio policies, see our Editorial Policies and the Editorial Policy Checklist.

Statistics

For all statistical analyses, confirm that the following items are present in the figure legend, table legend, main text, or Methods section.

n/a	Confirmed	
☐	☒	The exact sample size (*n*) for each experimental group/condition, given as a discrete number and unit of measurement
☐	☒	A statement on whether measurements were taken from distinct samples or whether the same sample was measured repeatedly
☐	☒	The statistical test(s) used AND whether they are one- or two-sided<br>*Only common tests should be described solely by name; describe more complex techniques in the Methods section.*
☒	☐	A description of all covariates tested
☒	☐	A description of any assumptions or corrections, such as tests of normality and adjustment for multiple comparisons
☐	☒	A full description of the statistical parameters including central tendency (e.g. means) or other basic estimates (e.g. regression coefficient) AND variation (e.g. standard deviation) or associated estimates of uncertainty (e.g. confidence intervals)
☐	☒	For null hypothesis testing, the test statistic (e.g. *F*, *t*, *r*) with confidence intervals, effect sizes, degrees of freedom and *P* value noted<br>*Give P values as exact values whenever suitable.*
☒	☐	For Bayesian analysis, information on the choice of priors and Markov chain Monte Carlo settings
☒	☐	For hierarchical and complex designs, identification of the appropriate level for tests and full reporting of outcomes
☒	☐	Estimates of effect sizes (e.g. Cohen's *d*, Pearson's *r*), indicating how they were calculated

Our web collection on statistics for biologists contains articles on many of the points above.

Software and code

Policy information about availability of computer code

Data collection	No software was used
Data analysis	Microsoft Excel<br>GraphPadPrism 5<br>Adobe Photoshop (version 24)<br>FlowJo software<br>Zen2.6 pro<br>LabImage 1D software<br>DAVID Bioinformatics from National Institutes of Health<br>https://github.com/LandthalerLab/EAE_Otud7b<br>GraphPad Prism 10

For manuscripts utilizing custom algorithms or software that are central to the research but not yet described in published literature, software must be made available to editors and reviewers. We strongly encourage code deposition in a community repository (e.g. GitHub). See the Nature Portfolio guidelines for submitting code & software for further information.

Data

Policy information about availability of data

All manuscripts must include a data availability statement. This statement should provide the following information, where applicable:

- Accession codes, unique identifiers, or web links for publicly available datasets
- A description of any restrictions on data availability
- For clinical datasets or third party data, please ensure that the statement adheres to our policy

Spatial transcriptomics raw data generated in this study have been deposited in Gene Expression Omnibus (GEO) under the accession number GSE286422. Bulk RNA-sequencing data have been submitted under accession number GSE286263. Source data are provided with the paper.

Research involving human participants, their data, or biological material

Policy information about studies with human participants or human data. See also policy information about sex, gender (identity/presentation), and sexual orientation and race, ethnicity and racism.

Reporting on sex and gender	*Use the terms sex (biological attribute) and gender (shaped by social and cultural circumstances) carefully in order to avoid confusing both terms. Indicate if findings apply to only one sex or gender; describe whether sex and gender were considered in study design; whether sex and/or gender was determined based on self-reporting or assigned and methods used. Provide in the source data disaggregated sex and gender data, where this information has been collected, and if consent has been obtained for sharing of individual-level data; provide overall numbers in this Reporting Summary. Please state if this information has not been collected. Report sex- and gender-based analyses where performed, justify reasons for lack of sex- and gender-based analysis.*
Reporting on race, ethnicity, or other socially relevant groupings	*Please specify the socially constructed or socially relevant categorization variable(s) used in your manuscript and explain why they were used. Please note that such variables should not be used as proxies for other socially constructed/relevant variables (for example, race or ethnicity should not be used as a proxy for socioeconomic status). Provide clear definitions of the relevant terms used, how they were provided (by the participants/respondents, the researchers, or third parties), and the method(s) used to classify people into the different categories (e.g. self-report, census or administrative data, social media data, etc.) Please provide details about how you controlled for confounding variables in your analyses.*
Population characteristics	*Describe the covariate-relevant population characteristics of the human research participants (e.g. age, genotypic information, past and current diagnosis and treatment categories). If you filled out the behavioural & social sciences study design questions and have nothing to add here, write "See above."*
Recruitment	*Describe how participants were recruited. Outline any potential self-selection bias or other biases that may be present and how these are likely to impact results.*
Ethics oversight	*Identify the organization(s) that approved the study protocol.*

Note that full information on the approval of the study protocol must also be provided in the manuscript.

Field-specific reporting

Please select the one below that is the best fit for your research. If you are not sure, read the appropriate sections before making your selection.

[x] Life sciences   [ ] Behavioural & social sciences   [ ] Ecological, evolutionary & environmental sciences

For a reference copy of the document with all sections, see nature.com/documents/nr-reporting-summary-flat.pdf

Life sciences study design

All studies must disclose on these points even when the disclosure is negative.

Sample size	No statistical method was used to estimate the sample size. Sample size was determined based on previous studies in the field. (PMID: 30944096, PMID: 24077734, PMID: 23011975). A minimum of 3 mice per group were analyzed for flow cytometry, histology, immuno-fluorescence, histology, bulk RNA sequencing and Spatial transcriptomics. This number was sufficient to yield a high enough number of events to show statistical differences between the different experimental groups. Exact numbers are provided in figure legends. For qPCR a minimum of three technical replicates from 3 different mice per condition were performed ensuring enough datasets for statistical analysis.
Data exclusions	No data were excluded from analysis
Replication	All results provided in this study were reproduced with a minimum of 3 replicates.
Randomization	This is not relevant to this study. Here, we compared two groups: Otud7bfl/fl controls and GFAP-Cre Otud7bfl/fl animals, throughout the progression of the disorder. Age- and sex-matched animals were allocated to each group.
Blinding	Blinding was not required in any experiments as phenotypes were clear

Reporting for specific materials, systems and methods

We require information from authors about some types of materials, experimental systems and methods used in many studies. Here, indicate whether each material, system or method listed is relevant to your study. If you are not sure if a list item applies to your research, read the appropriate section before selecting a response.

Materials & experimental systems

n/a	Involved in the study
☐	☒ Antibodies
☒	☐ Eukaryotic cell lines
☒	☐ Palaeontology and archaeology
☐	☒ Animals and other organisms
☒	☐ Clinical data
☒	☐ Dual use research of concern
☒	☐ Plants

Methods

n/a	Involved in the study
☒	☐ ChIP-seq
☐	☒ Flow cytometry
☒	☐ MRI-based neuroimaging

Antibodies

Antibodies used

Reagent or Resource Source Identifier Source Dilution
Antibodies for Western blotting
α-OTUD7b Proteintech 16605-1-AP Rabbit 1:1000
α-GFAP Proteintech 16823-1-AP Rabbit 1:5000
α- β Tubulin Proteintech 10068-1-AP Rabbit 1:5000
α-GAPDH Cell Signaling Technology 2118 Rabbit 1:5000
α-phospho STAT1(Tyr 701) Cell Signaling Technology 9167 Rabbit 1:5000
α-phospho STAT1(Ser 727) Cell Signaling Technology 9177 Rabbit 1:1000
α-STAT1 Cell Signaling Technology 9172 Rabbit 1:5000
α-phospho-p65 Cell Signaling Technology 3031 Rabbit 1:1000
α-IκBα Cell Signaling Technology 4812 Rabbit 1:1000
α-p65 Cell Signaling Technology 8242 Rabbit 1:1000
α-phospho-p38 MAPK Cell Signaling Technology 9215 Rabbit 1:1000
α-p38 MAPK Cell Signaling Technology 9212 Rabbit 1:1000
α-phospho-p44/42 MAPK Cell Signaling Technology 9101 Rabbit 1:1000
α-p44/42 MAPK Cell Signaling Technology 9102 Rabbit 1:1000
α-TRAF2 Cell Signaling Technology 4724 Rabbit 1:1000
α-RIPK1 Cell Signaling Technology 3493 Rabbit 1:1000
α-phospho JNK Cell Signaling Technology 4668 Rabbit 1:1000
α-JNK Cell Signaling Technology 9252 Rabbit 1:1000
α-TNFAIP3/A20 Cell Signaling Technology 5630 Rabbit 1:1000
α-phospho STAT3 Cell Signaling Technology 9131 Rabbit 1:1000
α-STAT3 Cell Signaling Technology 9139 Mouse 1:5000
α-cIAP1 Abcam ab154525 Rabbit 1:1000
α-ubiquitin Lys48-specific Merck Millipore 05-1307 Rabbit 1:1000
α-ubiquitin Lys63-specific Merck Millipore 05-1308 Rabbit 1:1000
α-ubiquitin Lys11-specific Merck Millipore MABS-107-I Rabbit -
Rabbit α-mouse IgG/HRP Dako P0161 Rabbit -
Swine α-rabbit IgG/HRP Dako P0399 Swine -
Mouse α-rabbit IgG, light chain specific Jackson ImmunoResearch AB_2339149 Mouse -
Goat α-mouse IgG, light chain specific Jackson ImmunoResearch AB_2338512 Goat -
Flourochrome conjugated antibodies for flow cytometry
α-CD3e-APC-Cy7 BioLegend 100204, 17A2 Rat 1:100
α-CD3e-PE eBioscience 12-0033-82, REA975 Hamster 1:100
α-CD4-BV421 BioLegend 100443, GK1.5 Rat 1:100
α- CD8α-APC eBioscience 17-0081-82, 53-6.7 Rat 1:100
α-CD8α-FITC eBioscience 11-0081-85, 53-6.7 Rat 1:100
α-CD45-PerCP BioLegend 103130, 30-F11 Rat 1:100
α-CD11b-PE-Cy7 BioLegend 101216, M1/70 Rat 1:100
α-F4/80-BV421 BioLegend 123132, BM8 Rat 1:100
α-Ly6C-APC eBioscience 17-5932-82, HK1.4 1:100
α-Ly6G-PE BioLegend 127608, 1A8 Rat 1:100
α-CD19-PE eBioscience 12-0193-82, 1D3 Rat 1:100
α-CD45R/B220-BV421 BD Bioscience 562922, RA3-6B2 Rat 1:100
α-CD11c-PE eBioscience 12-0114-83, N418 Hamster 1:100
α-ACSA-2-PE eBioscience 130-123-284, IH3-18A3 Rat 1:100

	α-IFN- γ eBioscience 12-7311-82, XMG1.2 Rat 1:100<br>α-IL-17 BioLegend 506904, TC11-18H10.1 Rat 1:100<br>α-GM-CSF BioLegend 505406, MP1-22E9 Rat 1:100<br>Primary antibodies for Immunohistochemistry and histology<br>α-GFAP Agilent AB_2811722 Rabbit 1:1000<br>α-Sox2 Abcam AB_10710406 Mouse 1:500<br>α-Sox9 RnD Systems AB_2194160 Mouse 1:500<br>α-Iba-1 Abcam AB_283346 Rat 1:800<br>α-GFAP Dako GA52461-2 Rabbit 1:1000<br>DAPI Thermo Fischer Scientific D1306 -<br>Alexa488-conjugated donkey anti-goat Thermo Fischer Scientific AB_2534102 Donkey -<br>Alexa488-conjugated donkey anti-mouse Thermo Fischer Scientific AB_141607 Donkey -<br>Cy3-conjugated donkey anti-rabbit Jackson AB_2340607 Donkey -<br>Cy5-conjugated donkey anti-rabbit Jackson AB_2340607 Donkey -
Validation	All the antibodies used are commercially available and the information is available on the manufacturer's website

Animals and other research organisms

Policy information about studies involving animals; ARRIVE guidelines recommended for reporting animal research, and Sex and Gender in Research

Laboratory animals	Otud7b fl/fl and GFAP-Cre Otud7bfl/fl mice were used in this study and all mice were bred on a C57BL/6J background. 8-12 weeks old animals were used for the study. Mice were housed in specific pathogen free enviorment. Animals were exposed to a 12-12h light/dark cycle, maintained at a regulated temperature and humidity and food and water was provided ad libitum.
Wild animals	No wild animals were used in this study
Reporting on sex	Both male and female mice were used in this study.
Field-collected samples	This study did not include field-collected samples
Ethics oversight	All animal experiments were in compliance with the German Animal Welfare Act in a protocol approved by the state authorities (Landesverwaltungsamt Sachsen Anhalt). File number 42502-2-1260.

Note that full information on the approval of the study protocol must also be provided in the manuscript.

Plants

Seed stocks	*Report on the source of all seed stocks or other plant material used. If applicable, state the seed stock centre and catalogue number. If plant specimens were collected from the field, describe the collection location, date and sampling procedures.*
Novel plant genotypes	*Describe the methods by which all novel plant genotypes were produced. This includes those generated by transgenic approaches, gene editing, chemical/radiation-based mutagenesis and hybridization. For transgenic lines, describe the transformation method, the number of independent lines analyzed and the generation upon which experiments were performed. For gene-edited lines, describe the editor used, the endogenous sequence targeted for editing, the targeting guide RNA sequence (if applicable) and how the editor was applied.*
Authentication	*Describe any authentication procedures for each seed stock used or novel genotype generated. Describe any experiments used to assess the effect of a mutation and, where applicable, how potential secondary effects (e.g. second site T-DNA insertions, mosiacism, off-target gene editing) were examined.*

Flow Cytometry

Plots

Confirm that:

- [x] The axis labels state the marker and fluorochrome used (e.g. CD4-FITC).
- [x] The axis scales are clearly visible. Include numbers along axes only for bottom left plot of group (a 'group' is an analysis of identical markers).
- [x] All plots are contour plots with outliers or pseudocolor plots.
- [x] A numerical value for number of cells or percentage (with statistics) is provided.

Methodology

Sample preparation	Described in materials and methods section
Instrument	Cytek Northern Light Flow Cytometer

Software	FlowJo
Cell population abundance	*Describe the abundance of the relevant cell populations within post-sort fractions, providing details on the purity of the samples and how it was determined.*
Gating strategy	The gating strategy is provided as Supplementary figure 1E

☒ Tick this box to confirm that a figure exemplifying the gating strategy is provided in the Supplementary Information.
