# Supplementary material for: Astrocytic-OTUD7B ameliorates murine experimental autoimmune encephalomyelitis by stabilizing glial fibrillary acidic protein and preventing inflammation
Source: Nat Commun. 2025 Oct 20;16:9279. doi: 10.1038/s41467-025-65093-4 (PMC12537900; doi:10.1038/s41467-025-65093-4)
Supplement: Supplementary file 8 — Supplementary Source Data [file 41467_2025_65093_MOESM8_ESM.docx]

**Uncropped scans for all blots**

Supplementary Figure 1B

GAPDH

OTUD7b


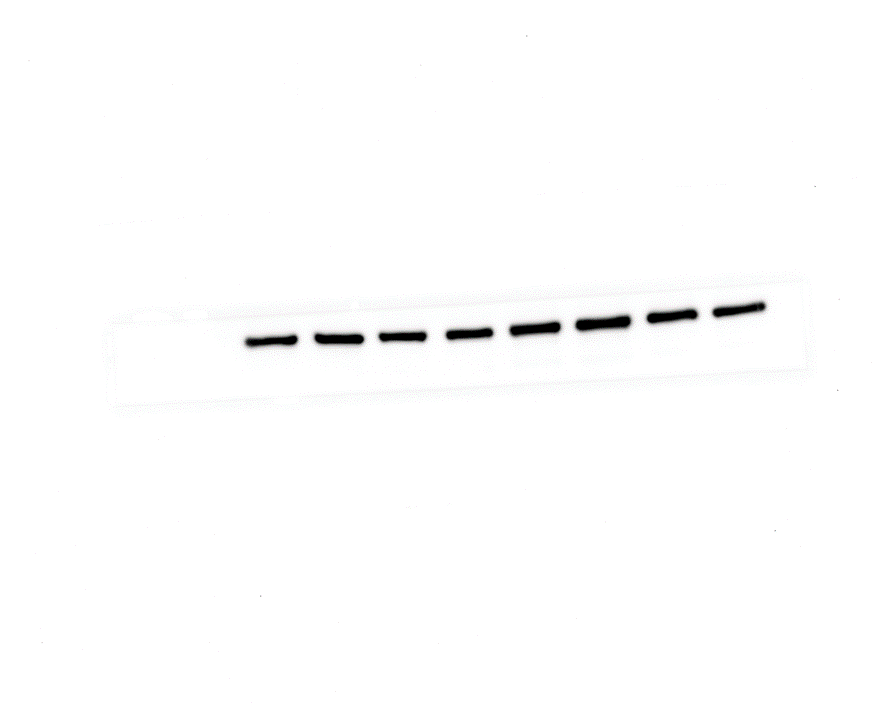

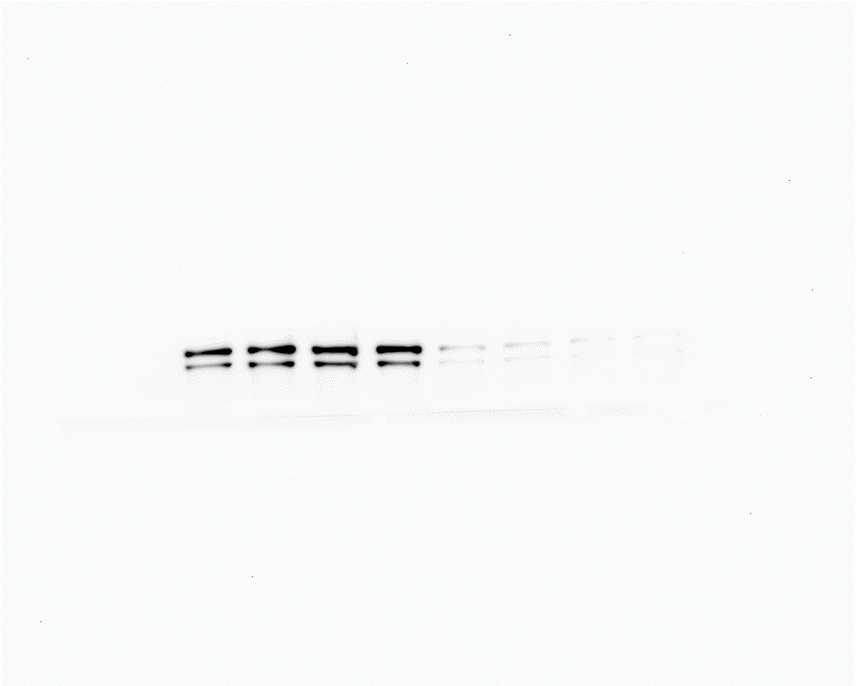


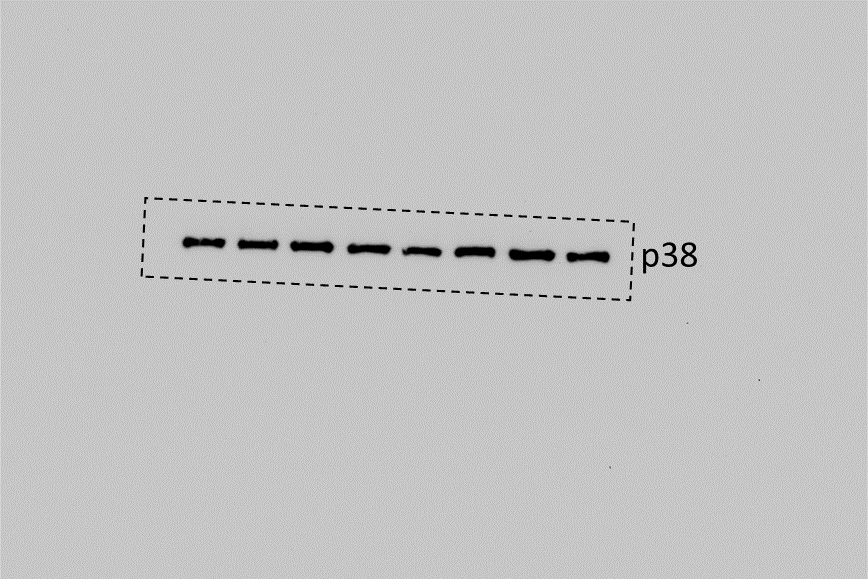

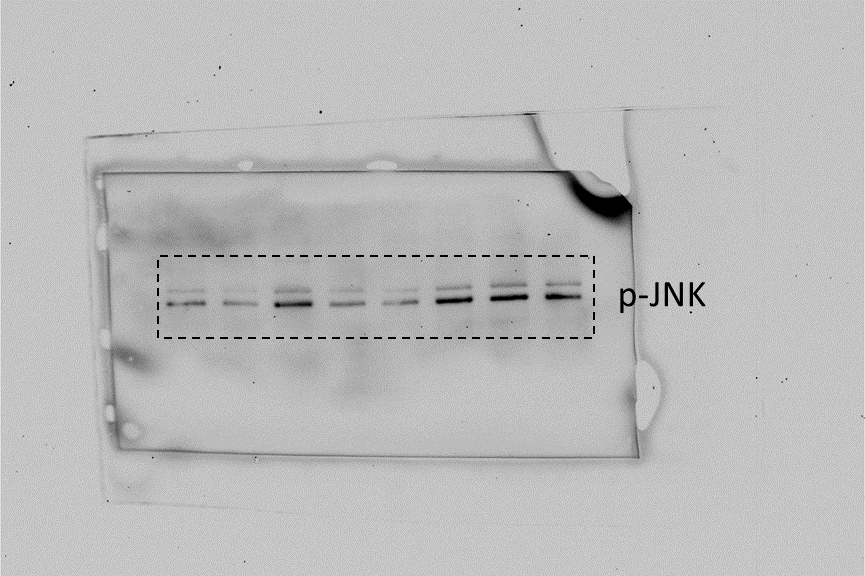

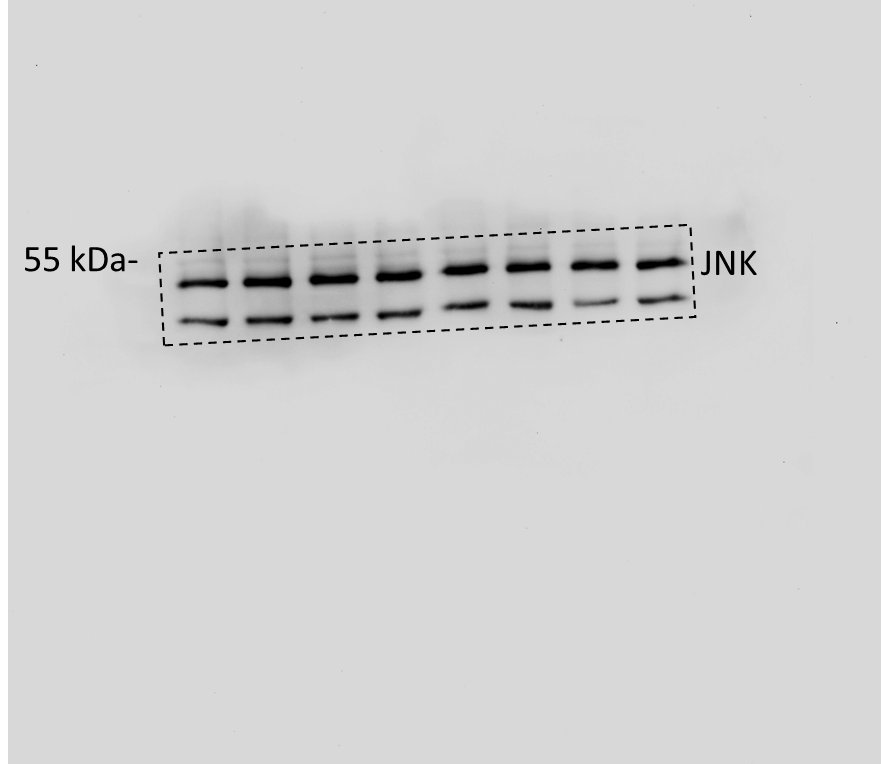

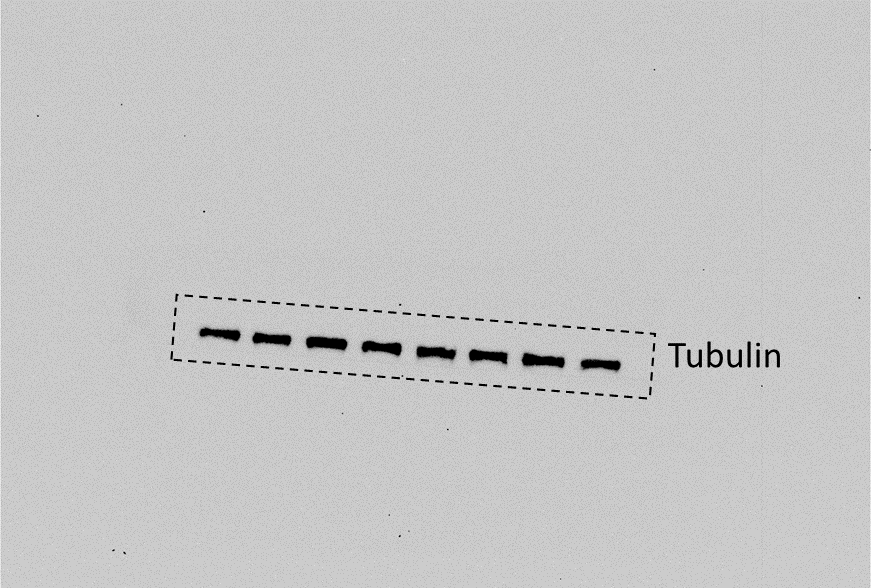

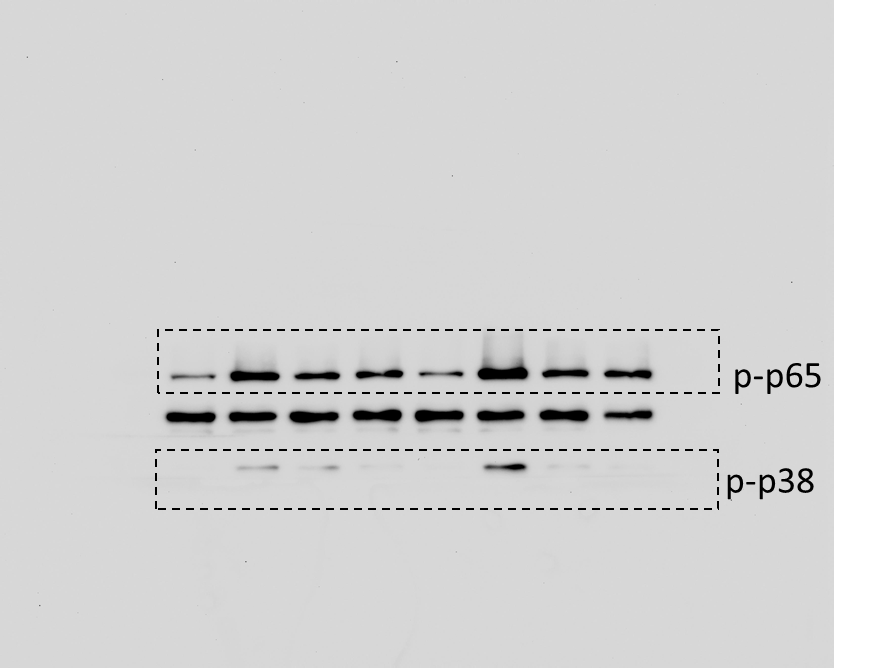

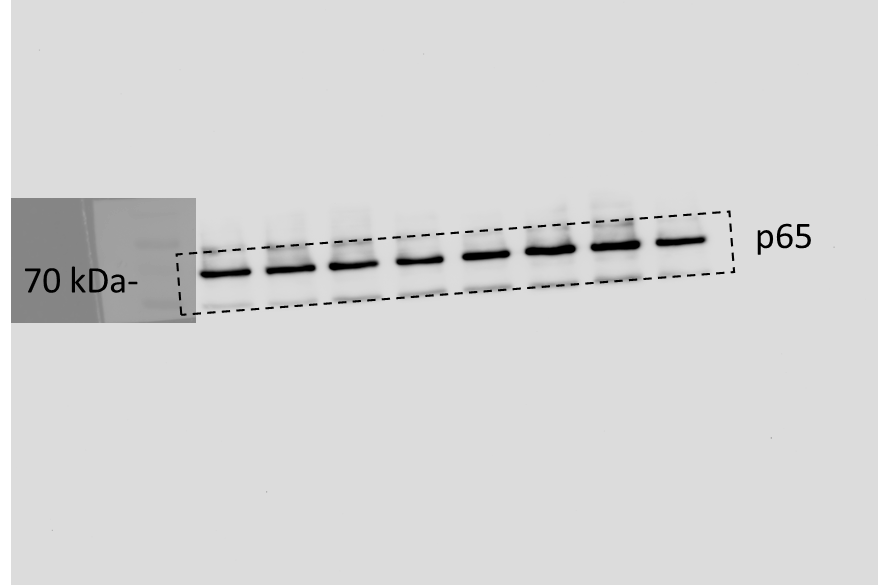

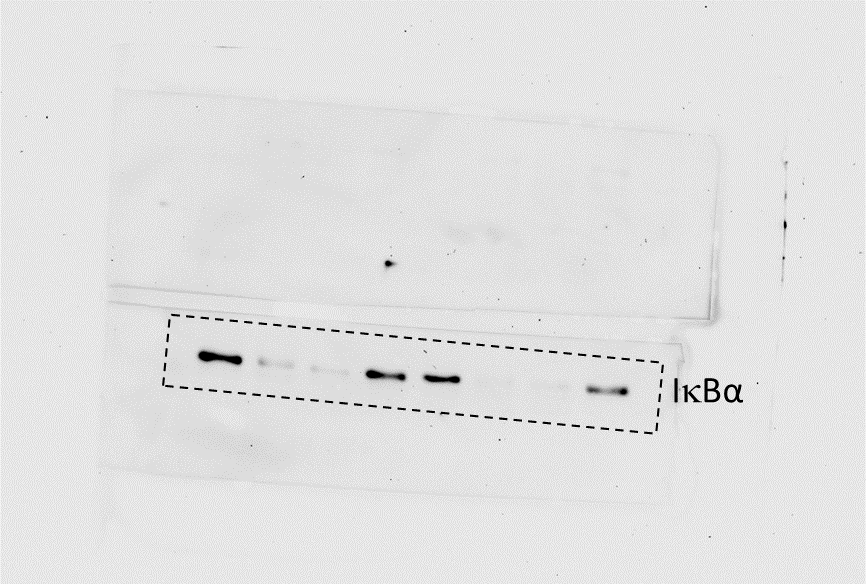

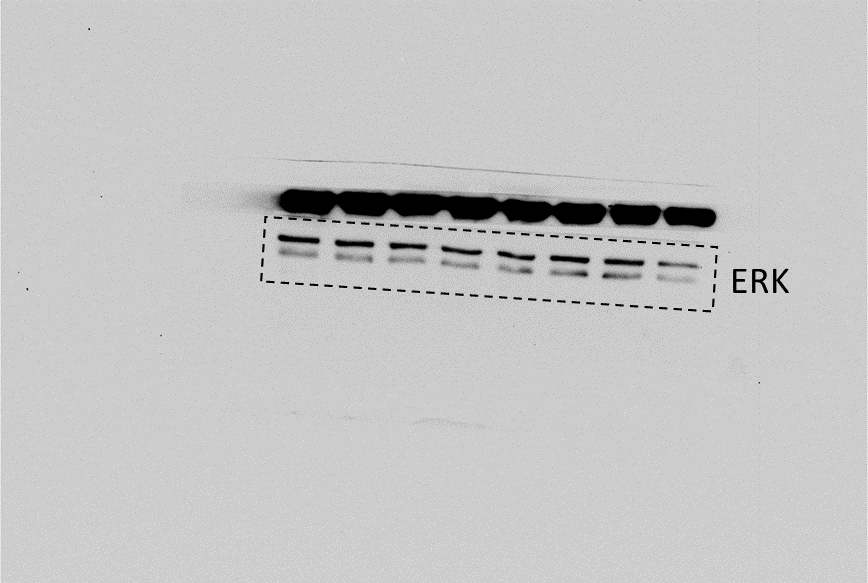

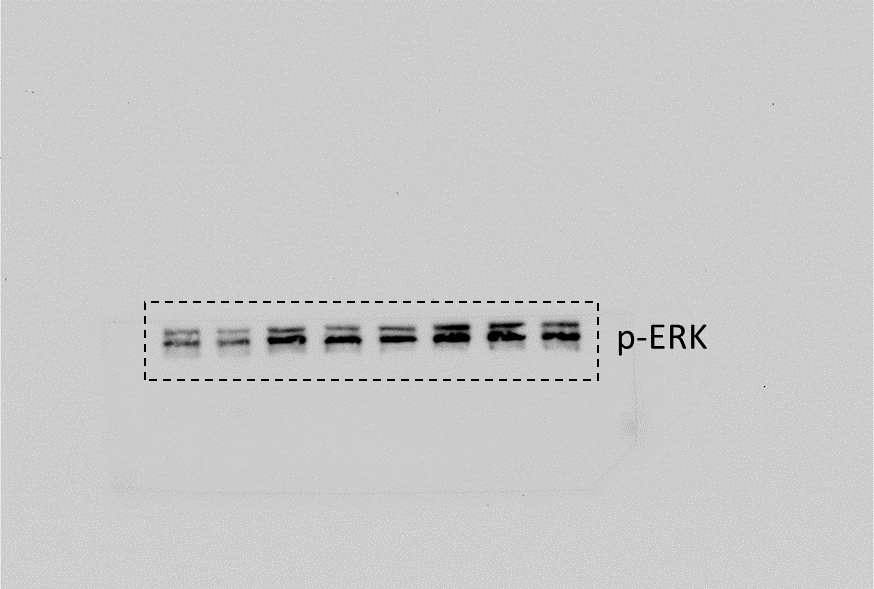
 Supplementary figure 2B

Supplementary figure 2A

**
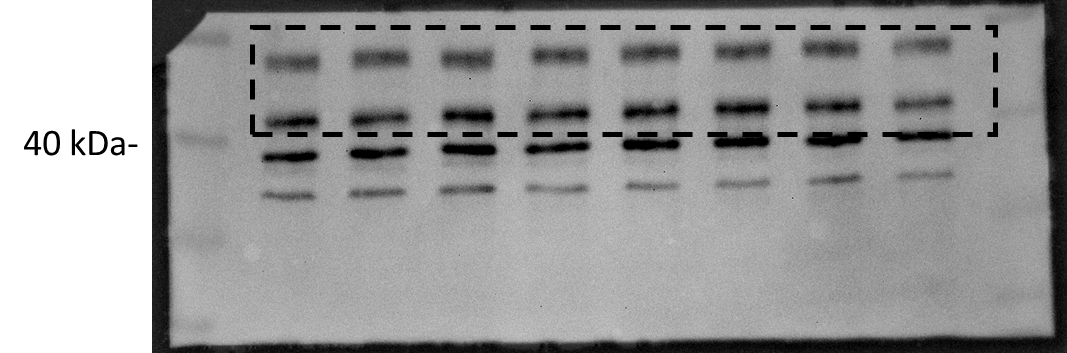

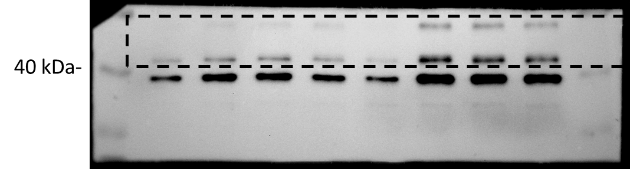

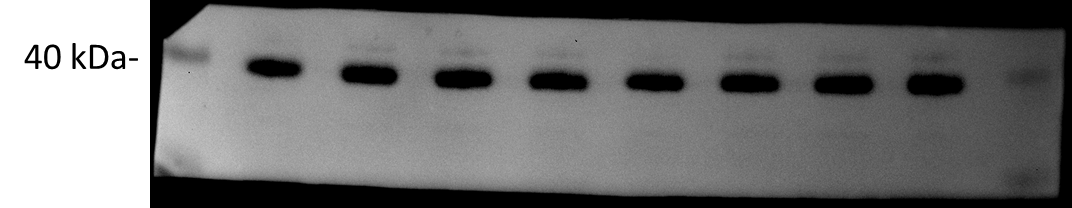

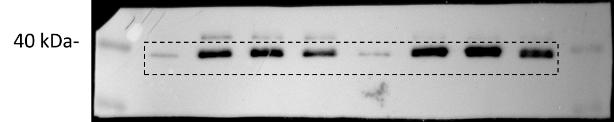

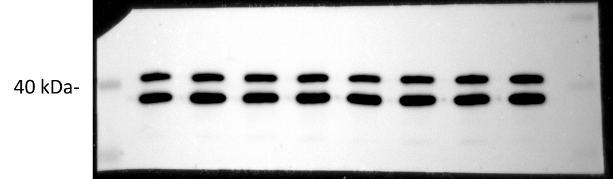

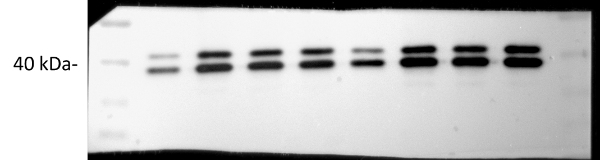

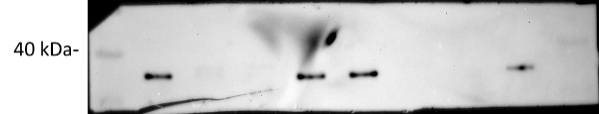

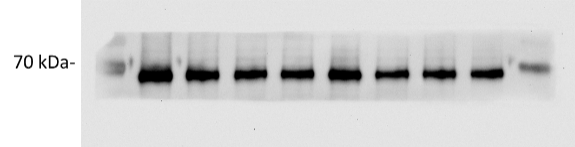

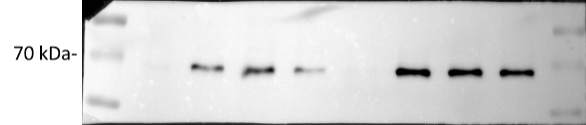
**

Tubulin

JNK

pJNK

p38

Pp38

ERK

p-ERK

IκBα

p65

Pp65

**
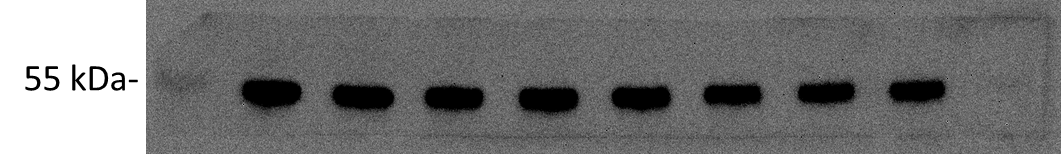
**

**
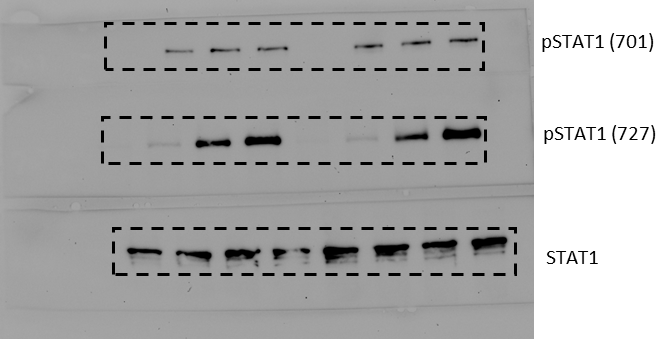
**

Supplementary figure 2D

**
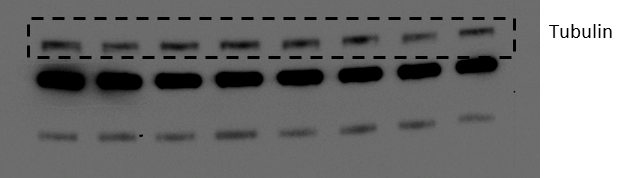

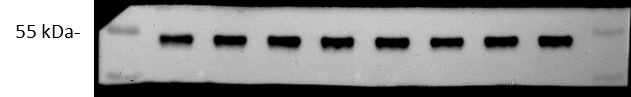

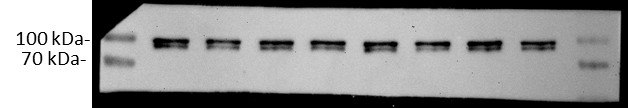

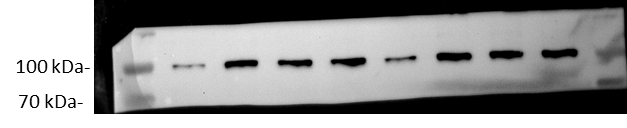

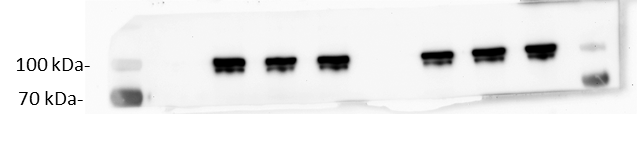
**

Supplementary figure 2C

Tubulin

STAT1

pSTAT1 (727)

pSTAT1 (701)

**
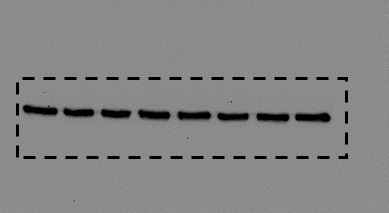

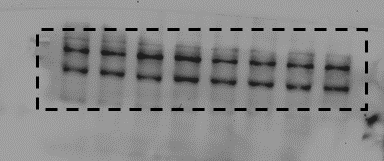

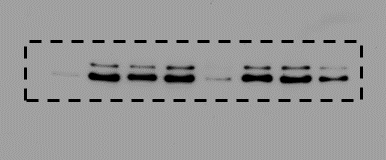

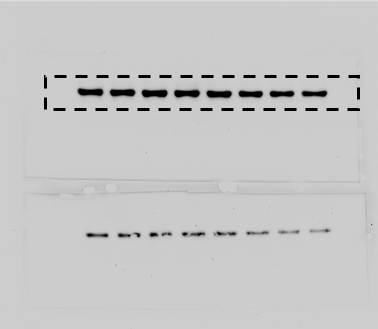

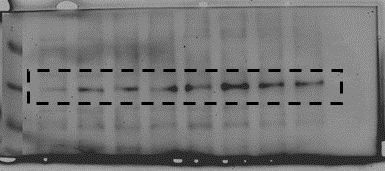

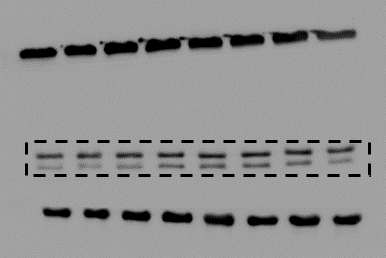

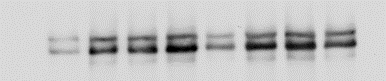

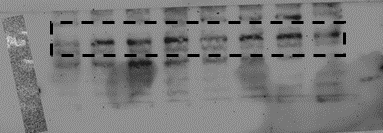

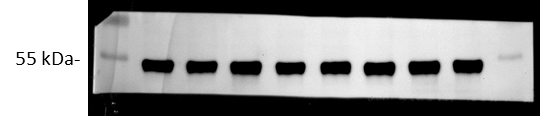

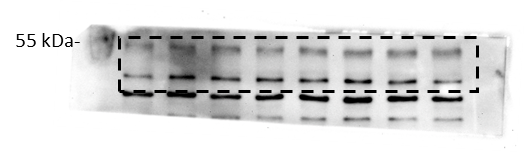

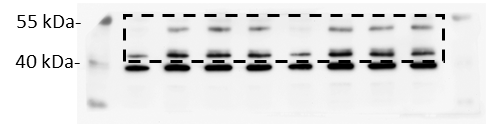

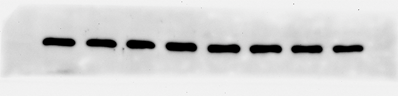

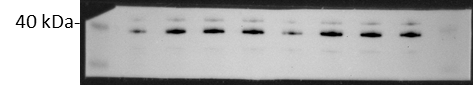

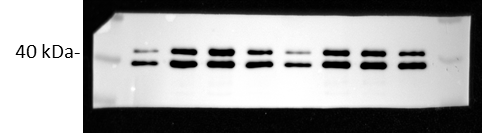

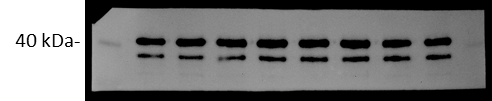

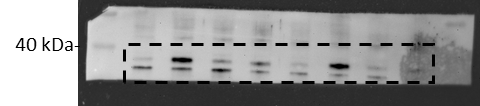
**

Tubulin

JNK

pJNK

p38

p-p38

Tubulin

JNK

pJNK

p38

p-p38

ERK

p-ERK

p-IκBα

Supplementary figure 2F

Supplementary figure 2E

p-IκBα

p-ERK

ERK

Supplementary figure 3A

**
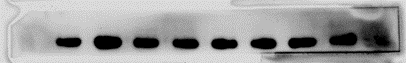

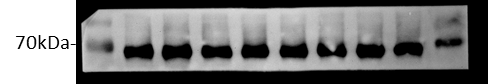

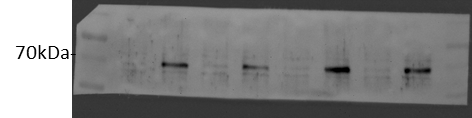

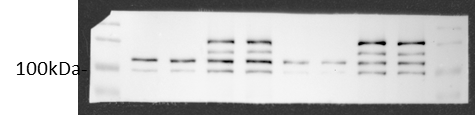

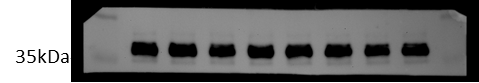

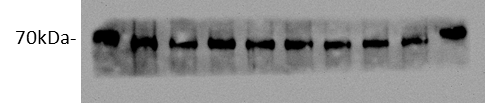

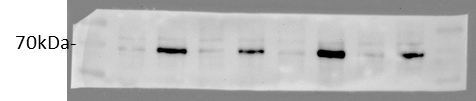

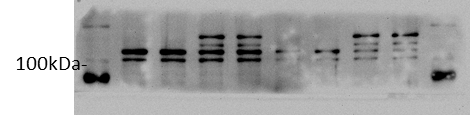
**

GAPDH

p65

p-p65

OTUD7b

GAPDH

p65

p-p65

OTUD7b

Supplementary figure 3B

**
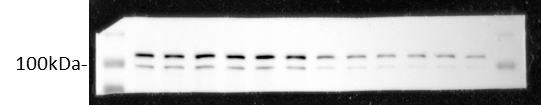
**

Supplementary figure 4B

Supplementary figure 4A

**
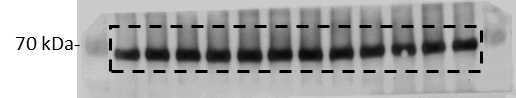

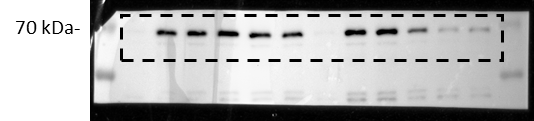

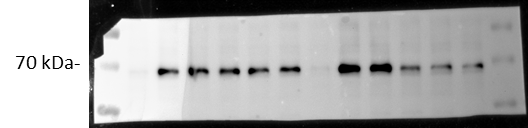

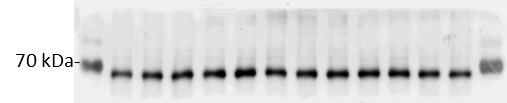

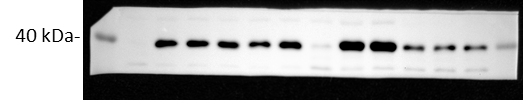

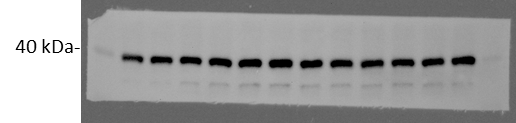

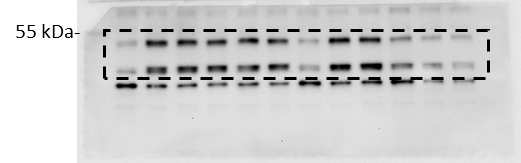

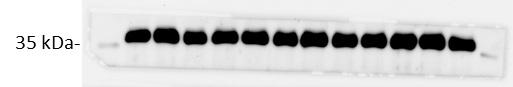

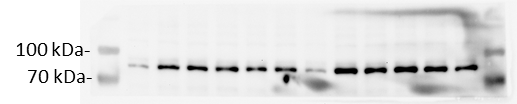

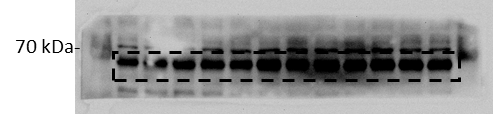

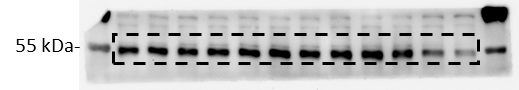

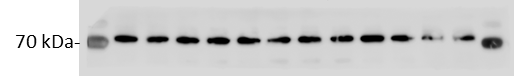

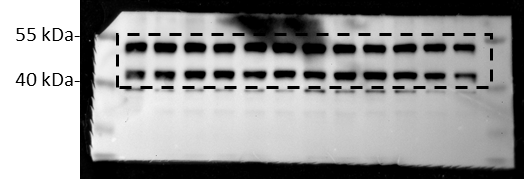

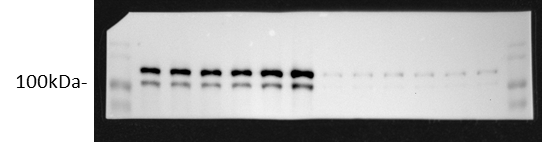

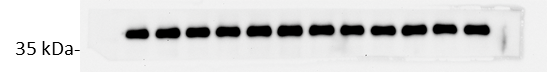

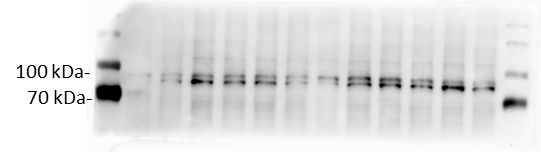

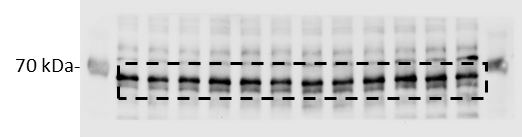

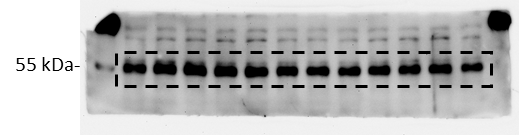

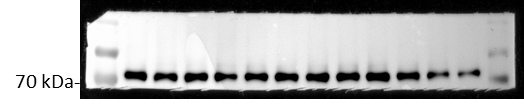

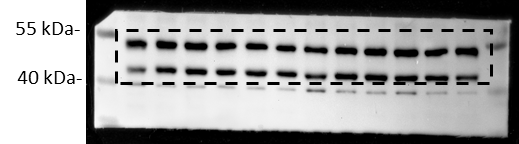

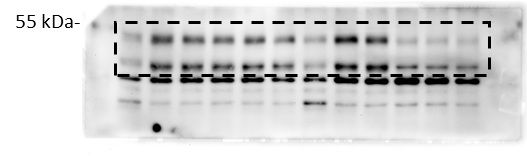

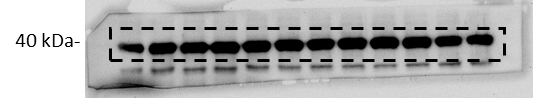

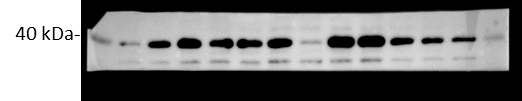
**

A20

GAPDH

GAPDH

cIAP1

TRAF2

RIPK1

JNK

pJNK

p38

pp38

p65

pp65

pp65

p65

pp38

p38

pJNK

A20

cIAP1

TRAF2

JNK

RIPK1

OTUD7b

OTUD7b

GAPDH

A20

Supplementary figure 4D

Supplementary figure 4C

**
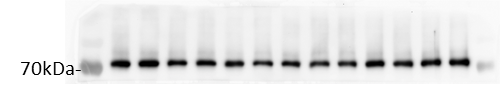

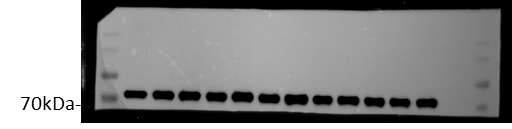

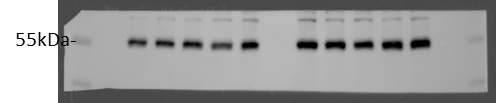

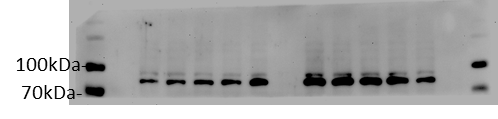

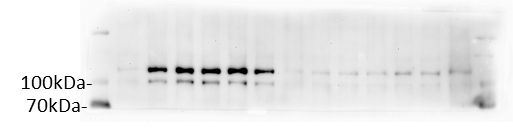

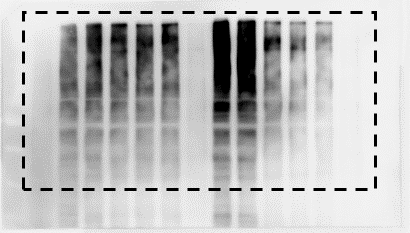

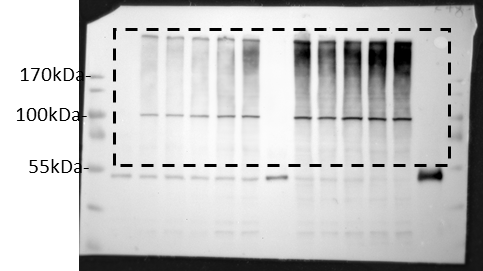

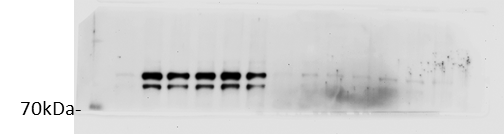

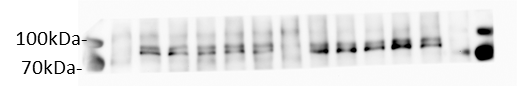

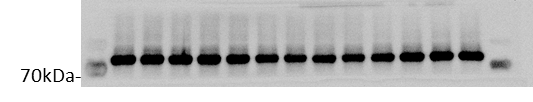

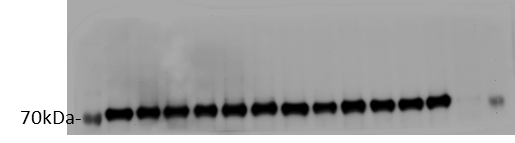

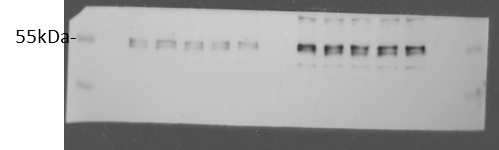

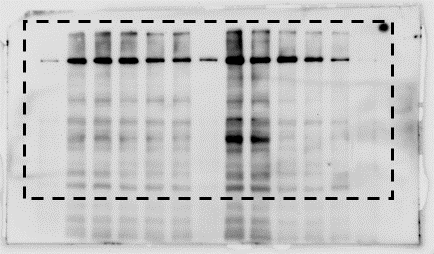

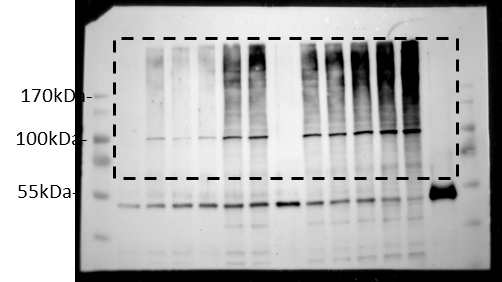
**

Input RIPK1

RIPK1

TRAF2

A20

OTUD7b

K63 Ubiquitination

K48 Ubiquitination

OTUD7b

A20

TRAF2

RIPK1

Input RIPK1

K63 Ubiquitination

K48 Ubiquitination

**
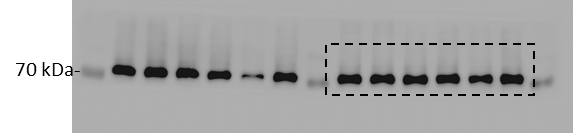

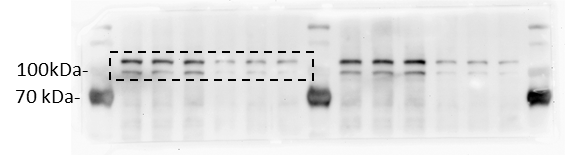
**

Supplementary figure 4E

RIPK1

Supplementary figure 5A

**
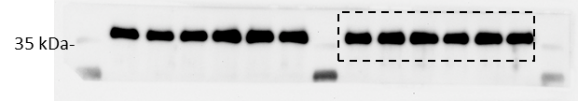

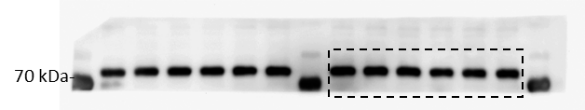

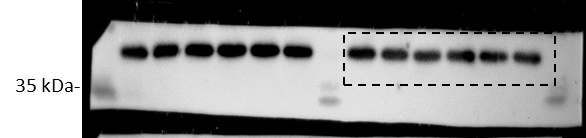

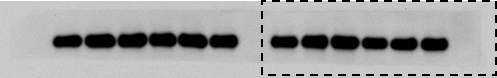

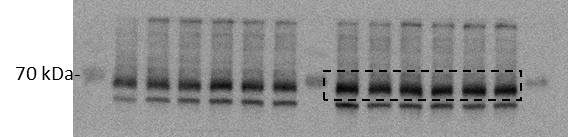

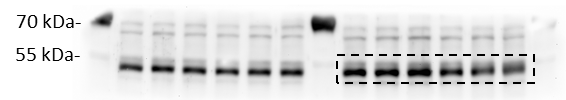

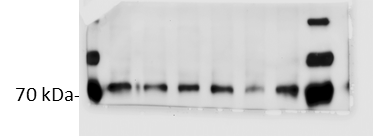

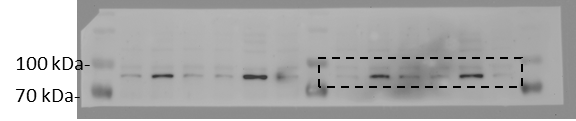

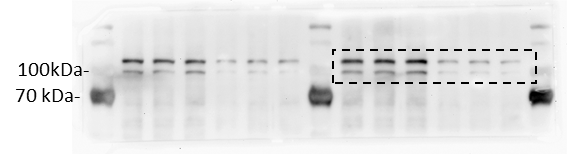
**

GAPDH

RIPK1

Supplementary figure 5B

GAPDH

GAPDH

cIAP1

TRAF2

RIPK1

Otud7b

A20

Supplementary figure 4F

TRAF2

cIAP1

GAPDH

RIPK1

A20

Otud7b

Supplementary figure 5D

Supplementary figure 5C

GAPDH

A20

JNK

pJNK

p38

p65

pp65

pp38

TRAF2

RIPK1

cIAP1

Otud7b

GAPDH

A20

JNK

pJNK

p38

pp38

p65

pp65

TRAF2

cIAP1

Otud7b

RIPK1

Supplementary figure 5F

Supplementary figure 5E

Input RIPK1

IP RIPK1

TRAF2

A20

OTUD7b

K63 Ubiquitination

K48 Ubiquitination

Input RIPK1

IP RIPK1

TRAF2

OTUD7b

A20

K63 Ubiquitination

K48 Ubiquitination

Supplementary figure 6B

Supplementary figure 6A

GAPDH

GFAP

GFAP

GAPDH

Supplementary figure 6D

Supplementary figure 6C

GAPDH

GFAP

STAT3

p-STAT3

OTUD7b

GAPDH

GFAP

STAT3

p-STAT3

OTUD7b

Supplementary figure 6F

Supplementary figure 6E

Input GFAP

GFAP

OTUD7b

K48 Ubiquitination

Input GFAP

GFAP

OTUD7b

K48 Ubiquitination
